# The hospital emigration to another region in the light of the environmental, social and governance model in Italy during the period 2004-2021

**DOI:** 10.1186/s12889-024-19369-x

**Published:** 2024-07-15

**Authors:** Emanuela Resta, Onofrio Resta, Alberto Costantiello, Angelo Leogrande

**Affiliations:** 1 University of Foggia, Foggia, Puglia Italia; 2 University of Bari Aldo Moro, Bari, Puglia Italia; 3 LUM University Giuseppe Degennaro, Strada Statale 100 km 18, Casamassima, Bari, Puglia Italia; 4 LUM Enterprise s.r.l. Strada Statale 100 km 18, Casamassima, Bari, Puglia Italia

**Keywords:** Hospital emigration, Regional inequalities, Panel data, Instrumental variable estimation, Machine-learning, I11, I12, I13, I14, I15, I18

## Abstract

The following article presents an analysis of the impact of the Environmental, Social and Governance-ESG determinants on Hospital Emigration to Another Region-HEAR in the Italian regions in the period 2004-2021. The data are analysed using Panel Data with Random Effects, Panel Data with Fixed Effects, Pooled Ordinary Least Squares-OLS, Weighted Least Squares-WLS, and Dynamic Panel at 1 Stage. Furthermore, to control endogeneity we also created instrumental variable models for each component of the ESG model. Results show that HEAR is negatively associated to the E, S and G component within the ESG model. The data were subjected to clustering with a k-Means algorithm optimized with the Silhouette coefficient. The optimal clustering with k=2 is compared to the sub-optimal cluster with k=3. The results suggest a negative relationship between the resident population and hospital emigration at regional level. Finally, a prediction is proposed with machine learning algorithms classified based on statistical performance. The results show that the Artificial Neural Network-ANN algorithm is the best predictor. The ANN predictions are critically analyzed in light of health economic policy directions.

## Introduction

The following article analyses the topic of patient mobility within the Italian regions. The analyzed data were acquired from ISTAT-BES. ISTAT-BES refers to the principles of the Sustainable Development Goals-SDGs. The ISTAT-BES variables have been reworked to highlight the three components E-Environmental, S-Social, and G-Governance of the ESG model. The issue of hospital migration of patients is becoming increasingly relevant in the Italian regions. In fact, the Italian regions are characterized by significant gaps from the point of view of per capita income, and also by the degree of socio-cultural sophistication of the production organizations and institutions operating at a regional level. It follows that there is a continuous violation of the principle of equality of Italian citizens in access to health services. This socio-economic and institutional condition leads Italian citizens to experience forms of hospital migration. The issue of patient mobility could become increasingly relevant in the future due to the presence of strong independence and autonomist movements present in the northern regions as in the case of the Northern League, which are calling for a true secession between Lombardy, Veneto and Emilia Romagna and the rest of the country . It must be considered that hospital migration is not only due, in general, to economic-social issues, but also to issues of quality and supply of healthcare services. In fact, the regions of Northern Italy tend to offer better health services and push southern citizens to migrate for health reasons. Finally, this migration increasingly concerns not only patients, but also doctors and nurses who move from the South to the North to have better career and work opportunities.

Furthermore, it must be considered that the interregional hospital migration of southern patients towards Northern Italy also produces a significant transfer of financial resources from the poor regions of Southern Italy to the rich regions of Northern Italy, increasing the level of territorial inequality in access to the healthcare system. Finally, healthcare migration is part of a broader macro-phenomenon detected at a national level, namely the low level of healthcare spending as a percentage of GDP, especially when compared with the similar levels of the most efficient European countries such as France and Germany. The resulting picture is therefore of a health system which has strongly desired the regionalization of the provision of health services, and which has nevertheless failed to guarantee equality in access to health services, causing significant social, economic and financial costs both for the citizens and for the regional institutions that must reimburse patient mobility. The sparsely populated regions such as Basilicata and Molise deserve a special case apart. In fact, for these regions it is very difficult to organize universal healthcare that can fully correspond to the needs of the population. In fact, the small Italian regions tend to be at the top in terms of hospital migration. The future of Italian healthcare is therefore very uncertain, both due to the presence of strong tensions on the fiscal autonomy of the northern regions, and due to the insufficiency of financial resources dedicated to the healthcare system of the southern regions. It is probable that in the absence of future interventions to restructure the healthcare system, the healthcare inequality between the southern regions and the northern regions will become increasingly accentuated with a significant impact in terms of quality of life.

Furthermore, hospital migration offers further incentives to stimulate healthcare investment in the Northern regions and disinvestment in the Southern regions. In the future, therefore, the gap between North and South, in terms of access to healthcare, could be unbridgeable, also due to the migration of doctors and nurses from South to North.

The article continues as follows: in the second section we present the relevant scientific literature, in the third section we present a comparative analysis of hospital migration at a European level, in the fourth section we present the data, in the fifth section we analyze the methodologies for selecting the variables of the econometric model, in the sixth section we analyze the scientific methodology applied for the metric analysis, in the seventh section we analyze the econometric models for estimating ESG impacts in determining hospital migration, the eighth section presents the clustering with optimized k-Means algorithm with the Silhouette coefficient, in the ninth section we present a prediction through a comparison with machine learning algorithms aimed at estimating the future value of hospital migration in the Italian regions, the tenth section analyses the international relevance of the analysis of hospital migration in the Italian regions, the eleventh section concludes.

## Literature review

Below is an analysis of the literature that takes into consideration patient migration in Europe and in Italy. However, there is also a nod to patient migration in China's large populous cities. Finally, what according to the literature are the motivations that push patients to seek better care in other regions or other countries are highlighted. In general, however, we can underline that patient migration tends to be a widespread phenomenon within nations between areas that have different health service facilities. However, patient mobility at an international level remains marginal.

### Patient mobility in Europe

The migration of patients between various European states for healthcare reasons raises both ethical and financial questions. There are ethical issues related to the need to guarantee all European citizens access to the best care even when it can be administered in other countries. There are also financial issues, as countries that have advanced healthcare systems must also bear the healthcare cost of offering treatment to European citizens from less fortunate countries [43]. Despite the existence of European directives that facilitate cross-border migration for health and treatment reasons, data shows that cases of patient migration at an intra-European level remain scarce [9]. Patient tourism, which is common in Asia and the United States, is also becoming widespread in Europe. However, the structure of this market is still uncertain, especially as regards the presence of agencies specialized in organizing patient tourism trips. Furthermore, absolutely new for the European market is the possibility of a mobility of medical-health personnel as a result of the development of health tourism [22]. The possibility for European patients to be treated in any European country regardless of their country of residence raises important issues that need to be resolved. In particular, there are legal, financial, administrative and organizational issues that must be resolved to enable efficient intra-community hospital migration. To this end, the creation of a European institution with control and coordination purposes could allow European citizens to be more likely to effectively use the right to healthcare mobility in Europe [12]. The presence of private hospitals and the possibility of increasing patient mobility at a European level increases the efficiency of public healthcare systems. It is in fact possible to notice, thanks to the privatization of hospitals and healthcare migration, a reduction in waiting lists and a reduction in healthcare costs [6]. Patient mobility in France at an interregional level is increased between 2014 and 2019 from 22.9% to 24.6%. The possibility of patent mobility between European nations poses legal and financial issues that have only been partially resolved with European legislation and the rulings of the European judiciary [51, 60, 62, 97]. Patient mobility in Spain is positively associated to income and the quality of health services [20]. The mobility of doctors and nurses is greater than patient mobility in Europe, even if only patient mobility has been considered relevant in the European legislation [46]. Service availability, poor quality and the regulatory systems are the main drivers of patient mobility in regional and public oriented healthcare systems i.e. Italy and Spain [80]. Patients move from low income regions to high-income regions in Turkey, to have access to better healthcare [83]. Patient mobility is very low considering among Norway, Sweden, Denmark and Finland [55].

### Patient mobility in Italy

Patient mobility tends to grow in connection with the severity of healthcare conditions. For this reason, it is necessary to calculate the incidence of extreme health phenomena in order to calculate the capacity of hospitals and regional health systems to take care of hospital migration. It is therefore possible to connect the capacity of hospitals to offer healthcare services with the structured demand that brings together both the regional and interregional dimensions to calculate the efficiency of healthcare facilities [72]. Lombardy is a leading region in healthcare emigration [73]. Patients move from southern to northern regions. Patient mobility also has the consequence of transfer financial resources from poor regions to rich regions [42]. In the medium to long term, the regions of southern Italy may be incapable of offering adequate health services to the population by promoting patient mobility toward the North, in the absence of an equalization intervention by the central government [30].

### Intercity patient mobility

In the case of very populous cities, such as in China for example, it is possible to verify an inefficient geographical distribution of hospital structures and services compared to the population. It is possible to use machine-learning tools to efficiently model the allocation of hospital resources within the territory of cities to best meet the needs of the population [34].

### Patient mobility choice

Patient mobility can depend on a series of socio-economic reasons or also connected to the ability of health systems to offer services corresponding to users' needs. Patient mobility tends to grow with the reduction of waiting lists, the growth in the quality of services, and access to advanced technologies. Patient mobility decreases with age and socio-economic status [2]. Considering hospital migration in the Italian regions, it is possible to note that one of the reasons that push patients to migrate is hospital specialization and the performance of neighbouring regions. Furthermore, the choice to emigrate for health reasons also depends on income factors, performance and technology [10]. There are four reasons that support the choice of hospital migration, namely: lower financial costs, possibility of also having access to complementary services not offered in the place of residence, improvement in the quality of care, offer of public financial resources to support healthcare migration. These motivations are common in a comparative study of the USA, Mexico and Europe [63]. Location of physicians can have a relevant role in determining patent mobility [59]. There is a positive relationship between patent mobility and the reduction of waiting times [14]. Patient mobility increases with the reduction of costs and the increase in the level of competition among providers, as a study in six European countries shows [47]. The development of geographical areas specializing in the provision of health services can be associated with the development of the patent mobility sector at a global level [68].

## Patient mobility across Europe: a comparative analysis

Patient mobility in Europe is primarily regulated by Directive 2011/24/EU, which establishes the rights of EU citizens to access healthcare services in any member state and to be reimbursed by their home country. This directive facilitates cross-border healthcare, allowing patients to seek medical treatments abroad, especially in cases of prolonged waiting times or when specific treatments are unavailable in their home country. According to data from the European Commission, the main reasons patients travel abroad for healthcare include access to specialist treatments, reduction of waiting times, and seeking high-quality care. Statistics show an expansion in the types of treatments that can be obtained without prior authorization, such as care for pre-existing conditions that worsen while abroad. Moreover, patient mobility presents both medical and legal challenges, such as the compatibility of reimbursement systems and continuity of care. Studies indicate that, despite the legal framework allowing a certain fluidity, significant barriers remain, including the complexity of reimbursement procedures and differences in healthcare quality levels across countries. These data highlight the importance of raising awareness of patient rights and optimizing administrative procedures to ensure more equitable and effective access to cross-border healthcare in Europe [40].

According to the most recent data, in 2022 there were significant movements of patients within the EU. Approximately 200,000 reimbursement requests were handled under the directive, with total expenditures exceeding 50 million euros. The countries with the highest number of outbound patients include Germany, France, and the Netherlands, while the most common destinations were Belgium, Spain, and Austria. In 2021, more than 150,000 EU citizens received medical care abroad without prior authorization, taking advantage of the possibility to obtain reimbursements for treatments not readily available in their home countries. The main treatments sought abroad included specialized surgical interventions and advanced therapies not available locally or with long waiting times. These data underscore the importance of cross-border cooperation in healthcare to ensure that all EU citizens can access high-quality care promptly, reducing disparities between various national healthcare systems [40].

Hospital mobility requests are divided into applications that require prior authorization and applications that do not require prior authorization. Therefore, below we will analyze the quantitative and financial characteristics of both types of requests in the context of European legislation and practice.

### Hospital mobility applications requiring prior authorization

Analyzing the 2022 data on hospital mobility in European countries reveals significant insights into the patterns and trends of cross-border healthcare within the EU. The dataset outlines the number of hospital care requests received, authorized, and refused by various European countries, along with the respective percentages of authorized and refused requests. The figures shed light on the operational efficiency, accessibility, and cooperation within the European healthcare framework. The total number of requests across all listed countries was 4,552, with 3,653 authorized and 840 refused, resulting in an overall authorization rate of 80.25% and a refusal rate of 18.45%. This high authorization rate suggests a robust mechanism for cross-border healthcare within the EU, though the refusal rate indicates room for improvement in ensuring all citizens have equal access to healthcare services abroad. Belgium received 47 requests, authorizing 12.77% and refusing 87.23%. The high refusal rate could indicate strict criteria or comprehensive local healthcare services that reduce the need for authorizations. Bulgaria had 4 requests with no authorizations and a 75% refusal rate. This data may reflect limited infrastructure for handling cross-border healthcare or a lack of outgoing patient mobility. Denmark received 41 requests, authorizing 17.07% and refusing 70.73%. Similar to Belgium, Denmark’s higher refusal rate might point to stringent approval processes or sufficient local healthcare services reducing the necessity for treatment abroad. Germany stands out with 2,781 requests, of which 85.47% were authorized and 14.53% refused. This high authorization rate demonstrates Germany's facilitation of cross-border healthcare, possibly due to its advanced healthcare system and efficient administrative processes. Ireland reported no requests, authorizations, or refusals, indicating either a complete reliance on local healthcare services or possible underreporting. Greece received 4 requests, with an equal split of 50% authorized and 50% refused. This balanced outcome suggests moderate efficiency in handling cross-border healthcare requests. Spain authorized 85.71% of its 7 requests, with a refusal rate of 14.29%. Spain's data mirrors Germany's high authorization rate, reflecting effective cross-border healthcare management. Croatia had 3 requests with a 33.33% authorization rate and 66.67% refusal rate. The relatively high refusal rate might indicate barriers in administrative processes or limited capacity for cross-border healthcare. Italy received 76 requests, authorizing 72.37% and refusing 27.63%. Italy’s authorization rate is notably high, suggesting efficient cross-border healthcare processes. Luxembourg processed 942 requests, authorizing 67.73% and refusing 32.27%. Given its small size, Luxembourg’s significant number of requests and high authorization rate reflect its pivotal role in European healthcare mobility. Hungary authorized none of its 3 requests, resulting in a 100% refusal rate. This could indicate stringent criteria or inefficiencies in the cross-border healthcare process. Malta had 6 requests, all authorized, reflecting a 100% authorization rate. Malta’s performance suggests an exceptionally efficient handling of cross-border healthcare requests. Poland had 1 request, which was refused, indicating potential barriers in accessing cross-border healthcare. Portugal authorized all 7 of its requests, reflecting a 100% authorization rate. Similar to Malta, Portugal demonstrates an effective system for cross-border healthcare. Romania received 4 requests, with a 25% authorization rate and no refusals. This unique situation suggests that some requests might be pending or require additional processing. Slovenia had 47 requests, authorizing 23.40% and refusing 38.30%. The significant percentage of refused requests may indicate procedural inefficiencies or limited cross-border healthcare resources. Slovakia processed 579 requests, with a high authorization rate of 92.57% and a low refusal rate of 3.45%. Slovakia’s data reflects an efficient system for cross-border healthcare management [40]. The results are shown in Table 1 below:

**Table 1:** Hospital mobility applications requiring prior authorization

Country	Received	Authorised	Refused	% Authorised	% Refused
Belgio	47	6	41	12.77	87.23
Bulgaria	4	0	3	0	75
Denmark	41	7	29	17.07	70.73
Germany	2781	2377	404	85.47	14.53
Ireland	0	0	0	0	0
Greece	4	2	2	50	50
Spain	7	6	1	85.71	14.29
Croatia	3	1	2	33.33	66.67
Italy	76	55	21	72.37	27.63
Luxembourg	942	638	304	67.73	32.27
Hungary	3	0	3	0	100
Malta	6	6	0	100	0
Poland	1	0	1	0	100
Portugal	7	7	0	100	0
Romania	4	1	0	25	0
Slovenia	47	11	18	23.4	38.3
Slovakia	579	536	20	92.57	3.45
Total	4552	3653	840	80.25	18.45

Countries like Germany, Spain, and Italy show high authorization rates, reflecting their robust healthcare systems and efficient handling of cross-border healthcare requests. Countries like Malta and Portugal, despite receiving fewer requests, demonstrate 100% authorization rates, indicating exceptionally efficient cross-border healthcare processes. Countries like Hungary, Bulgaria, and Poland show high refusal rates or no authorizations, indicating potential challenges in cross-border healthcare infrastructure or administrative processes. Despite its size, Luxembourg processed a significant number of requests with a high authorization rate, highlighting its important role in the European healthcare landscape. The absence of data from countries like Ireland suggests possible gaps in data reporting or unique healthcare dynamics that limit the need for cross-border care. The analysis of hospital mobility data in European countries for 2022 reveals significant insights into the efficiency and challenges of cross-border healthcare within the EU. While many countries demonstrate high authorization rates, indicating effective systems, others face challenges that need addressing to ensure equitable access to healthcare for all EU citizens. Enhanced cooperation, administrative efficiency, and infrastructure investments are essential to optimize cross-border healthcare and reduce disparities among member states.

Analyzing the data on hospital emigration reimbursements requiring prior authorization reveals significant insights into the changes and trends in healthcare mobility across several European countries between 2021 and 2022. This analysis covers reimbursement amounts, absolute variations, and percentage variations, highlighting the financial dynamics and shifts in patient mobility for specific countries. The total reimbursements for hospital emigration across the listed countries skyrocketed from €1,468,975 in 2021 to €7,708,041 in 2022, marking an absolute variation of €6,239,066 and a staggering percentage increase of 424.72%. Belgium experienced a significant decrease in reimbursements, dropping from €22,363 in 2021 to €11,880 in 2022, an absolute decrease of €10,483, equating to a -46.88% change. This reduction might indicate improved domestic healthcare services or stricter authorization processes for cross-border treatments. Denmark also saw a notable decline, with reimbursements falling from €119,097 in 2021 to €76,285 in 2022, a decrease of €42,812, or -35.95%. This trend could suggest enhancements in Denmark’s local healthcare provision or changes in patient preferences and healthcare policies. Germany stands out with a dramatic increase in reimbursements, soaring from €525,871 in 2021 to €6,822,642 in 2022, representing an absolute rise of €6,296,771 and a percentage increase of 1197.40%. This extraordinary growth may reflect a significant surge in German patients seeking specialized treatments abroad or a response to backlog demands from the pandemic years. Greece also recorded a substantial increase, from €1,821 in 2021 to €46,865 in 2022, an absolute rise of €45,044 and a percentage change of 2473.59%. The low base in 2021 suggests that cross-border healthcare was previously minimal, but the sharp increase indicates a newfound reliance on or accessibility to foreign medical services. Spain experienced a significant reduction, with reimbursements dropping from €11,818 in 2021 to €5,700 in 2022, a decrease of €6,118, or -51.77%. This decline might be attributed to better domestic healthcare services or more stringent criteria for obtaining prior authorization for treatments abroad. Croatia reported an introduction of cross-border healthcare reimbursements in 2022 with €620, indicating the beginning of tracking or reimbursing such treatments. Italy saw a decrease from €200,485 in 2021 to €139,975 in 2022, a reduction of €60,510, or -30.18%. This trend could be due to improvements in the Italian healthcare system or reduced patient mobility due to lingering effects of the pandemic. Malta also experienced a reduction, from €79,695 in 2021 to €54,069 in 2022, a decrease of €25,626, or -32.16%. The decrease may suggest enhancements in local healthcare capabilities or stricter reimbursement policies. Slovenia reported an increase in reimbursements, rising from €14,523 in 2021 to €20,580 in 2022, an absolute increase of €6,057, or 41.71%. This rise could reflect a growing reliance on foreign healthcare services or increased access to cross-border treatments. Slovakia showed a moderate increase in reimbursements, from €493,302 in 2021 to €529,425 in 2022, a rise of €36,123, or 7.32%. This consistent increase indicates steady patient mobility for healthcare services abroad, possibly driven by specific treatment needs unavailable domestically. The overall increase in hospital emigration reimbursements suggests a broader trend of patients seeking healthcare solutions beyond their national borders, driven by various factors such as the need for specialized treatments, shorter waiting times, or higher quality care. Germany's exceptional increase might be due to its well-documented backlog of medical cases during the pandemic, where many patients delayed treatments and sought care abroad once travel restrictions eased. Conversely, countries like Belgium, Denmark, and Spain, which experienced significant decreases, may have improved their domestic healthcare services, thereby reducing the necessity for cross-border healthcare. Alternatively, these reductions could be due to policy changes making it harder to get authorization for treatments abroad. The case of Greece is particularly notable for its significant percentage increase, albeit from a low base, suggesting a critical reliance on cross-border healthcare which may reflect inadequacies or gaps in the local healthcare system. The results are shown in Table 2 below:

**Table 2:** Hospital emigration reimbursements requiring prior authorization

Country	2021	2022	Absolute Variation	Percentage Variation
Belgio	22.363,00 €	11.880,00 €	-10.483,00 €	-46.88
Danimarca	119.097,00 €	76.285,00 €	-42.812,00 €	-35.95
Germania	525.871,00 €	6.822.642,00 €	6.296.771,00 €	1197.4
Grecia	1.821,00 €	46.865,00 €	45.044,00 €	2473.59
Spagna	11.818,00 €	5.700,00 €	-6.118,00 €	-51.77
Croazia		620,00 €	620,00 €	
Italia	200.485,00 €	139.975,00 €	-60.510,00 €	-30.18
Malta	79.695,00 €	54.069,00 €	-25.626,00 €	-32.16
Slovenia	14.523,00 €	20.580,00 €	6.057,00 €	41.71
Slovacchia	493.302,00 €	529.425,00 €	36.123,00 €	7.32
Total	1.468.975,00 €	7.708.041,00 €	6.239.066,00 €	424.72

### Hospital mobility that does not require prior authorization

Analyzing the data on hospital mobility that does not require prior authorization provides a comprehensive view of the dynamics and trends in cross-border healthcare within Europe. The data presents the number of requests received, granted, and refused by different European countries, alongside the respective percentages of granted and refused requests. This analysis sheds light on the operational efficiency, accessibility, and variances in healthcare services across the region. The dataset reflects substantial variances in the number of requests received and the approval rates among different countries. The total requests range from as few as 8 in Bulgaria to as many as 300,254 in France, highlighting a significant disparity in patient mobility and cross-border healthcare needs. Countries like Estonia, Greece, Cyprus, Lithuania, and Malta exhibit exceptionally high approval rates, with Estonia and Malta showing near-perfect approval rates of 98.89% and 100%, respectively. This suggests that these countries have efficient administrative processes for handling cross-border healthcare requests, potentially due to smaller volumes of requests or more straightforward approval criteria. The high approval rates could also indicate robust bilateral agreements with neighbouring countries that facilitate smoother patient mobility. Germany and France stand out with the highest volumes of requests received—160,647 and 300,254, respectively. Germany granted 88.03% of its requests, reflecting its capacity to manage a large volume of cross-border healthcare efficiently. France, on the other hand, granted 84.40% but also showed a high refusal number of 111,383, which accounts for 37.10% of its total requests. This could point to a stringent evaluation process or capacity constraints in handling such a high volume of requests. Countries like Denmark, Italy, and Norway fall into the mid-range category concerning approval rates. Denmark received 23,415 requests, granting 77.04%, which indicates a relatively balanced approach to cross-border healthcare but also a significant refusal rate of 14.53%. Italy and Norway show similar trends with approval rates of 79.19% and 79.25%, respectively. These mid-range approval rates suggest that while these countries are open to cross-border healthcare, they maintain rigorous criteria for granting approvals. Ireland, Romania, and Latvia are notable for their lower approval rates. Ireland granted only 56.55% of its 1,841 requests, with a refusal rate of 5.49%. Romania and Latvia have approval rates of 57.59% and 26.32%, respectively, with relatively low refusal rates. These statistics might indicate inefficiencies in processing requests or stricter criteria for approval. The most striking case is Portugal, which did not grant any of its 27 requests, resulting in a 100% refusal rate. This indicates either an exceptionally stringent evaluation process or potential issues in bilateral healthcare agreements. Germany and France’s high volumes and high approval rates underscore their role as major hubs for cross-border healthcare within Europe. These countries likely have extensive healthcare infrastructure and robust administrative mechanisms to handle large volumes of patient mobility efficiently. Countries like Malta and Estonia, with smaller volumes of requests, can maintain near-perfect approval rates, reflecting efficient administrative processing and possibly fewer bureaucratic hurdles. Denmark, Italy, and Norway show a balanced approach with moderate volumes and mid-range approval rates, indicating a pragmatic approach to managing cross-border healthcare without overwhelming their healthcare systems. The data from countries like Portugal and Latvia, showing low volumes but high refusal rates, point towards potential barriers in administrative processes or restrictive healthcare policies that limit patient mobility. The data reveals that while some countries efficiently handle cross-border healthcare requests, others face challenges that may stem from administrative inefficiencies, restrictive policies, or limited bilateral agreements. Countries with lower approval rates should focus on streamlining their administrative processes to facilitate easier and faster approval of cross-border healthcare requests. Simplifying documentation requirements and enhancing digital processing can reduce bureaucratic delays. Enhancing bilateral healthcare agreements between countries can help improve approval rates and ensure patients have access to necessary treatments without extensive delays. These agreements should focus on mutual recognition of healthcare standards and simplified reimbursement procedures. Countries with high refusal rates might need to invest in capacity building within their healthcare systems to manage cross-border healthcare more effectively. This includes training healthcare administrators and improving infrastructure to support higher volumes of patient mobility. Raising awareness among patients about their rights and the processes involved in cross-border healthcare can help reduce the number of refusals due to incomplete or incorrect applications. Educational campaigns and easily accessible information portals can play a crucial role in this regard. Countries with stringent approval criteria should consider policy reforms to align more closely with the broader goals of the European Union’s cross-border healthcare directive. This includes adopting more flexible criteria for approval and ensuring that refusal reasons are clearly communicated and justified. The analysis of hospital mobility data for European countries that do not require prior authorization highlights significant disparities in how different nations handle cross-border healthcare. While some countries demonstrate high efficiency and approval rates, others face challenges that need to be addressed through administrative improvements, policy reforms, and enhanced bilateral cooperation. Ensuring equitable access to healthcare across borders remains a critical goal, and the insights from this data can guide targeted interventions to achieve a more integrated and efficient European healthcare system (Table 3).

**Table 3:** Refund applications that do not require prior authorization

Country	Received	Granted	Refused	% Granted	% Refused
Bulgaria	8	7	1	87.5	12.5
Repubblica Ceca	440	380	60	86.36	13.64
Danimarca	23415	18038	3402	77.04	14.53
Germania	160647	141411	19236	88.03	11.97
Estonia	90	89	1	98.89	1.11
Irlanda	1841	1041	101	56.55	5.49
Grecia	56	54	2	96.43	3.57
Spagna	9	8	1	88.89	11.11
Francia	300254	253419	111383	84.4	37.1
Croazia	192	123	69	64.06	35.94
Italia	149	118	31	79.19	20.81
Cipro	44	43	1	97.73	2.27
Lettonia	19	5	2	26.32	10.53
Lituania	131	125	6	95.42	4.58
Malta	10	10	0	100	0
Polonia	14176	11862	332	83.68	2.34
Portogallo	27	0	27	0	100
Romania	889	512	28	57.59	3.15
Slovenia	2190	1980	61	90.41	2.79
Slovacchia	13904	13161	723	94.66	5.2
Finlandia	5652			0	0
Svezia	16008	9077	831	56.7	5.19
Norvegia	7739	6133	1929	79.25	24.93

Analyzing the data on reimbursements for hospital migration that does not require prior authorization between 2021 and 2022 provides insightful perspectives on healthcare mobility trends within Europe. This analysis will consider the absolute and percentage variations in reimbursement amounts for each country, reflecting the financial shifts and underlying factors influencing patient mobility. The total reimbursements across all listed countries dropped significantly from €259,076,018 in 2021 to €86,462,491 in 2022, showing an absolute decrease of €172,613,527, which translates to a drastic percentage decrease of 66.63%. Belgium saw an increase in reimbursements from €6,448,551 in 2021 to €7,566,882 in 2022, marking an absolute variation of €1,118,331 or a 17.34% increase. This growth suggests a rising trend in patient mobility or an expansion in the scope of reimbursable treatments. Bulgaria recorded a reimbursement amount of €1,513 in 2022, reflecting its initiation into the recorded data. The absolute figure is relatively small, indicating limited patient mobility or healthcare needs requiring cross-border solutions. Czech Republic experienced a significant increase from €116,339 to €154,833, showing an absolute rise of €38,494 or 33.09%. This increase points to an enhanced utilization of cross-border healthcare services, possibly due to greater awareness or improved access. Denmark reported a rise in reimbursements from €2,522,780 to €2,836,897, an absolute increase of €314,117 or 12.45%. This moderate growth aligns with a steady demand for cross-border healthcare services. Germany had a remarkable surge in reimbursements, escalating from €5,953,279 in 2021 to €30,391,998 in 2022. The absolute increase of €24,438,719 corresponds to a substantial 410.51% growth. This dramatic rise could be attributed to a backlog of healthcare needs from the pandemic or significant policy changes facilitating easier access to cross-border treatments. Estonia saw its reimbursements jump from €71,000 to €238,000, an increase of €167,000 or 235.21%. This notable growth suggests a substantial increase in patients seeking healthcare abroad, possibly due to limitations in local healthcare services. Ireland faced a substantial reduction, with reimbursements falling from €7,811,328 to €4,323,231, an absolute decrease of €3,488,097 or -44.65%. This decrease could indicate improvements in domestic healthcare services, reducing the need for cross-border treatments. Greece experienced a decrease from €14,982 to €12,131, showing an absolute decline of €2,851 or -19.03%. This decline suggests better management of domestic healthcare needs or tighter controls on cross-border reimbursements. Spain showed a dramatic increase from €184 in 2021 to €7,197 in 2022, marking a €7,013 rise or 3811.41%. While the absolute numbers are small, the percentage increase indicates a growing trend in cross-border healthcare utilization. France experienced a significant drop in reimbursements from €214,292,169 to €14,123,939, a decrease of €200,168,230 or -93.41%. This drastic reduction could be due to policy changes, improved local healthcare capacity, or a reallocation of healthcare resources. Croatia saw an increase from €18,787 to €21,544, an absolute rise of €2,757 or 14.68%. This growth reflects a moderate increase in cross-border healthcare utilization. Italy exhibited a substantial rise in reimbursements from €48,222 to €102,571, an absolute increase of €54,349 or 112.71%. This significant growth suggests a higher reliance on cross-border healthcare solutions. Cyprus presented a new data entry with €379,964 in reimbursements, indicating its engagement in cross-border healthcare reimbursements. Latvia showed a reduction from €29,475 to €14,183, a decrease of €15,292 or -51.88%. This drop might be due to better domestic healthcare provisions or stricter reimbursement policies. Lithuania experienced an increase from €95,502 to €140,950, an absolute rise of €45,448 or 47.59%. This increase suggests a higher utilization of cross-border healthcare services. Malta faced a reduction from €15,270 to €10,724, showing a decrease of €4,546 or -29.77%. This decline indicates a reduced need for cross-border treatments. Poland reported an increase from €4,288,925 to €5,925,573, an absolute rise of €1,636,648 or 38.16%. This growth reflects a higher demand for healthcare services abroad. Romania showed an increase from €433,737 to €644,168, an absolute rise of €210,431 or 48.52%. This increase indicates a growing reliance on cross-border healthcare. Slovenia exhibited an increase from €483,147 to €793,879, an absolute rise of €310,732 or 64.31%. This significant growth suggests a higher utilization of cross-border healthcare services. Slovakia reported an increase from €1,372,334 to €2,741,784, an absolute rise of €1,369,450 or 99.79%. This near doubling indicates a substantial growth in cross-border healthcare needs. Finland showed an increase from €175,989 to €214,531, an absolute rise of €38,542 or 21.90%. This growth reflects a moderate increase in cross-border healthcare utilization. Sweden faced a reduction from €13,509,933 to €11,161,854, an absolute decrease of €2,348,079 or -17.38%. This decline might indicate improvements in domestic healthcare services. Norway exhibited a significant increase from €1,374,085 to €4,654,145, an absolute rise of €3,280,060 or 238.71%. This substantial growth suggests a higher reliance on cross-border healthcare services. The data reveals a mixed trend of increases and decreases in reimbursements for hospital migration across Europe. Countries like Germany, Estonia, and Norway show substantial increases, indicating a rising reliance on cross-border healthcare services. On the other hand, significant decreases in countries like France and Ireland suggest improved local healthcare capacities or changes in policy.

### Authorized requests for reimbursement by country of treatment

Analyzing the data on authorized requests for reimbursement by country of treatment provides a detailed view of patient mobility and the demand for healthcare services across different European countries. This analysis examines the number of authorized requests for each country, which reflects the extent to which patients are seeking and receiving cross-border healthcare services. The total number of authorized requests for reimbursement across all listed countries is 326,812. This figure highlights a substantial level of patient mobility within Europe, indicating significant interactions between national healthcare systems. France stands out with 258,729 authorized requests, which accounts for nearly 79% of the total requests. This dominant figure suggests that France is a major hub for cross-border healthcare within Europe, likely due to its advanced medical facilities, diverse range of treatments available, and possibly more efficient reimbursement processes. Denmark also shows a high volume of requests with 18,038, making it another key destination for cross-border healthcare. Denmark's healthcare system is known for its high standards and accessibility, which could explain the large number of patients seeking treatment there. Poland and Slovakia have 11,862 and 13,161 authorized requests, respectively. These figures indicate that these countries are significant destinations for cross-border healthcare, possibly due to cost-effective treatment options or specialized services not readily available in neighbouring countries. Sweden and Norway report 9,077 and 6,132 authorized requests, respectively. The relatively high numbers for these countries suggest robust healthcare systems that attract a considerable number of cross-border patients, possibly for specialized treatments or high-quality care. Finland with 5,632 authorized requests, and Ireland, with 709, reflect moderate levels of cross-border patient mobility. These figures indicate that these countries provide important healthcare services that attract patients from other regions.

Several countries show lower volumes of authorized requests, such as: Czech Republic 380, Estonia 89, Greece 54, Spain 8, Croatia 123, Italy 128, Cyprus 44, Latvia 15, Lithuania 125, Malta 7, Romania 512, Slovenia 1,980. These numbers suggest that while these countries do participate in cross-border healthcare, the demand for treatment there is relatively lower compared to major hubs like France and Denmark. This could be due to a variety of factors, including the availability of specialized treatments, the overall quality of healthcare, and patient preferences. The exceptionally high number of authorized requests for France suggests it is a preferred destination for many patients seeking treatment abroad. The country's healthcare system is likely perceived as offering high-quality and comprehensive medical care. The high numbers for Denmark, Poland, and Slovakia indicate regional preferences and possibly ease of access for neighbouring countries. Patients might choose these countries for their proximity, lower costs, or specific medical expertise. Countries like Finland, Sweden, and Norway, with moderate volumes, might be emerging as preferred destinations for specific types of treatments or healthcare services. The lower numbers for countries like Spain, Italy, and Greece might reflect either a lower demand for cross-border healthcare or sufficient domestic healthcare services that meet the needs of their populations. The data on authorized requests for reimbursement by country of treatment reveals significant trends and patterns in cross-border healthcare within Europe. With France leading as a major destination, and other countries like Denmark, Poland, and Slovakia also playing crucial roles, the landscape of patient mobility is complex and multifaceted. To optimize healthcare delivery and patient outcomes, it is essential for countries to focus on improving domestic healthcare services, streamlining administrative processes, and fostering cross-border cooperation. This approach will help ensure that all European citizens have access to high-quality healthcare, regardless of where they choose to receive treatment (Table 4).

**Table 4:** Authorized requests for reimbursement by country of treatment

Country	Authorised requests for reimbursement by country of treatment	Concentration %
France	258729	79.17
Denmark	18038	5.52
Slovakia	13161	4.03
Poland	11862	3.63
Spain	9077	2.78
Norway	6132	1.88
Finland	5632	1.72
Slovenia	1980	0.61
Ireland	709	0.22
Romania	512	0.16
Czeck Republic	380	0.12
Italy	128	0.04
Lithuania	125	0.04
Croatia	123	0.04
Estonia	89	0.03
Lithuania	54	0.02
Cypro	44	0.01
Latvia	15	0
Sweden	8	0
Bulgaria	7	0
Malta	7	0
Total	326812	100

## Data

### Data collection methods and sources

The ISTAT BES (Benessere Equo e Sostenibile) database is a significant initiative by the Italian National Institute of Statistics, aimed at measuring the well-being of the Italian population through a multidimensional perspective that goes beyond traditional economic indicators such as GDP. The primary goal of this database is to provide a more comprehensive and nuanced view of social, economic, and environmental progress, taking into account a wide range of dimensions that reflect the quality of life and well-being of individuals. The BES is organized into various domains that include health, education, work, economic well-being, social relationships, politics and institutions, safety, subjective well-being, landscape and cultural heritage, environment, research and innovation, and quality of services. Each domain contains specific indicators that allow for the evaluation and monitoring of various aspects of well-being, thereby contributing to a more detailed and realistic understanding of human and social development. For instance, within the health domain, indicators include life expectancy at birth, infant mortality rates, and access to healthcare services, while in the education and training sector, indicators such as school dropout rates and the percentage of graduates are featured. These data are collected and updated periodically, ensuring a dynamic and current view of living conditions in the country. Access to the data is facilitated through the ISTAT website, which offers interactive tools for visualization and analysis, allowing citizens, researchers, policymakers, and sector operators to explore the information in depth. This availability of open data is crucial for promoting transparency, participation, and accountability in public policies, encouraging informed debate based on concrete evidence. For example, indicators of subjective well-being, such as life satisfaction and psychological well-being, provide a unique perspective on the perceived quality of life by individuals, thus integrating objective measurements with personal experiences. Additionally, the environmental dimension of the BES, which includes indicators like air quality and waste management, emphasizes the importance of sustainability and environmental protection for the future well-being of generations. Therefore, the BES not only provides a detailed picture of current conditions but also serves as a strategic tool for planning and implementing policies aimed at improving the overall well-being of the population. The inclusion of data at regional and municipal levels allows for disaggregation that highlights territorial inequalities, offering insights for targeted and personalized interventions. This approach makes it possible to identify areas that require more attention and to assess the effectiveness of implemented policies, thereby contributing to a continuous improvement process. The educational dimension of the BES, for example, helps monitor progress in reducing the educational gap and promoting quality education for all, while the focus on safety and social relationships underscores the importance of social cohesion and crime prevention. Furthermore, the integration of research and innovation among the BES domains underscores the crucial role of science and technology in supporting sustainable development and promoting innovative solutions to social and environmental challenges. Finally, the BES is also a powerful communication and awareness tool used to educate and mobilize civil society towards a more equitable and sustainable development model. This multidimensional and integrated approach to well-being not only enriches statistical knowledge but also stimulates critical reflection on how policies can genuinely improve people's lives, promoting a balance between economic growth, social equity, and environmental sustainability. In this context, the BES represents a fundamental pillar for more informed and responsible governance, capable of addressing global and local challenges with an inclusive and forward-looking vision.

### The relationship between the ISTAT-BES database and the ESG model

The ISTAT-BES (Benessere Equo e Sostenibile) database provides a comprehensive and multidimensional framework for measuring the well-being and sustainable development of the Italian population, making it a valuable resource for analyzing ESG factors. ESG dynamics, which focus on the environmental, social, and governance aspects of sustainability, align closely with the various domains and indicators present in the ISTAT-BES database. This alignment allows for a detailed and nuanced understanding of how different factors contribute to sustainable development and overall well-being. The environmental domain of the ISTAT-BES database includes indicators that are directly relevant to the 'E' in ESG. These indicators cover aspects such as air quality, waste management, energy consumption, and the protection of natural resources. By analyzing these indicators, stakeholders can assess the environmental impact of policies and practices, monitor progress towards sustainability goals, and identify areas where improvements are needed. For example, air quality indicators can provide insights into the effectiveness of measures to reduce pollution, while data on waste management can highlight the success of recycling programs and waste reduction initiatives. Such information is crucial for businesses, policymakers, and investors who are increasingly focused on environmental sustainability. The social domain of the ISTAT-BES database is rich with indicators that correspond to the 'S' in ESG. These indicators encompass a wide range of factors that influence social well-being, including health, education, employment, social relationships, and quality of life. By examining these indicators, it is possible to capture the social impact of various policies and initiatives. For instance, health indicators such as life expectancy and access to healthcare services can shed light on the effectiveness of public health interventions, while education indicators like school dropout rates and the percentage of graduates can inform strategies to improve educational outcomes. Additionally, indicators related to social relationships and subjective well-being provide valuable insights into the overall quality of life and social cohesion within communities. This information is essential for understanding the social dimensions of sustainability and ensuring that development efforts are inclusive and equitable. The governance domain, while not explicitly delineated in the ISTAT-BES database, can still be inferred from various indicators related to institutional quality, civic engagement, and political participation. These aspects are crucial for the G in ESG, as good governance practices are fundamental to achieving sustainable development. Indicators such as voter turnout, trust in institutions, and the quality of public services can provide a proxy for governance quality. By analyzing these indicators, stakeholders can assess the effectiveness of governance structures and identify areas where reforms are needed. Good governance practices, such as transparency, accountability, and civic participation, are essential for creating an enabling environment for sustainable development and for fostering trust between citizens and institutions. Moreover, the ISTAT-BES database's multidimensional approach allows for the integration of ESG dynamics across different domains. For example, the interplay between environmental sustainability and social well-being can be explored by analyzing how environmental policies impact public health or how social initiatives contribute to environmental conservation. Similarly, the relationship between governance and social outcomes can be examined by looking at how institutional quality affects access to education or healthcare. This holistic perspective is crucial for capturing the interconnectedness of ESG factors and for developing comprehensive strategies that address multiple dimensions of sustainability simultaneously. In conclusion, the ISTAT-BES database is a powerful tool for capturing ESG dynamics and supporting sustainable development. Its comprehensive and multidimensional framework provides valuable insights into the environmental, social, and governance aspects of well-being, making it a valuable resource for businesses, policymakers, investors, and other stakeholders. By leveraging the data provided by the ISTAT-BES database, it is possible to develop informed strategies, make evidence-based decisions, and contribute to a more sustainable and equitable future.

### Limitations in the usage of ISTAT-BES database

While the ISTAT-BES (Benessere Equo e Sostenibile) database offers a comprehensive and multidimensional framework for assessing the well-being of the Italian population, it is not without limitations. One of the primary limitations of the ISTAT-BES database is the issue of data granularity and coverage. Although the database provides extensive data across multiple domains, the level of detail may vary significantly across different indicators and geographic areas. For example, some indicators are available only at the national or regional level, making it challenging to assess local variations in well-being. This lack of fine-grained data can obscure important differences within regions or municipalities, potentially leading to an incomplete understanding of localized issues. Additionally, some indicators may be updated less frequently than others, leading to temporal inconsistencies that can affect trend analysis and the timely evaluation of policies. Another significant limitation is the potential for data quality and reliability issues. The data in the ISTAT-BES database is derived from various sources, including surveys, administrative records, and other statistical methodologies. These sources may have inherent biases, sampling errors, or inconsistencies that can affect the accuracy and reliability of the indicators. For example, self-reported data on subjective well-being or social relationships can be influenced by personal biases or cultural factors that may not be uniformly represented across different demographic groups. Similarly, administrative data may suffer from underreporting or misclassification issues. Ensuring high data quality and addressing these potential biases requires rigorous validation and standardization procedures, which may not always be feasible or adequately implemented. Another limitation is the challenge of integrating qualitative and quantitative data. The ISTAT-BES database primarily relies on quantitative indicators to measure well-being, which can overlook the nuanced and context-specific aspects of quality of life that qualitative data can provide. While quantitative data is essential for standardized measurement and comparison, qualitative insights are crucial for understanding the underlying reasons behind the trends and patterns observed in the data. Incorporating qualitative data, such as personal narratives, case studies, or community consultations, can enrich the analysis and provide a more holistic understanding of well-being. However, this integration poses methodological challenges and requires innovative approaches to data collection and analysis. While the ISTAT-BES database is a powerful tool for measuring well-being and supporting sustainable development, it is important to recognize and address its limitations. Issues related to data granularity, quality, and representation, the integration of qualitative insights, the complexity of well-being dimensions, the adaptability to changing contexts, and the accessibility and usability of the database represent important limitations in using the database.

## Methodology for selecting model variables

To choose the variables to integrate within the model we used statistical methods. First of all we started by analyzing 122 variables from the ISTAT BES database. The 122 variables are divided into categories, namely:

- 10 variables in the education and training category;
- 13 variables in the work and life balance category;
- 9 variables in the economic well-being category;
- 9 variables in the social relations category;
- 10 variables in the politics and institutions category;
- 10 variables in the public safety category;
- 4 variables in the subjective well-being category;
- 11 variables in the landscape and cultural heritage category;
- 18 variables in the environment category;
- 10 variables in the innovation, research and creativity category;
- 16 variables in the public services category.

We then grouped the categories to arrive at the three categories of interest, namely E-Environment, S-Social and G-Governance. That is to say:

- E= environment + landscape and cultural heritage;
- S= education and training + work and life balance+ economic well-being+ social relations + subjective well-being;
- G= politics and institutions + public safety+ innovation, research and creativity + public services.

**Fig. 1:**
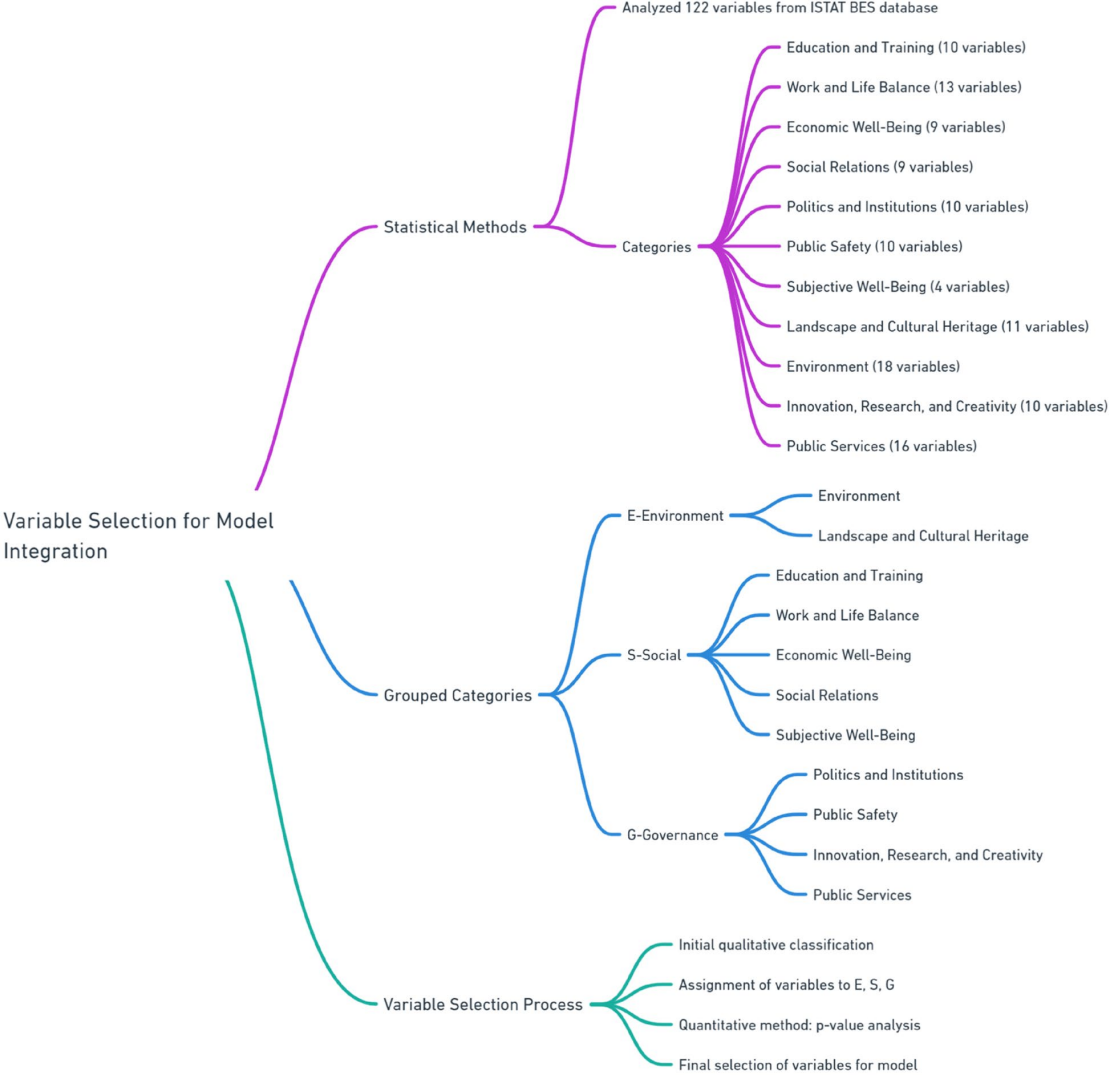
Summary of the methodology used to choose the variables of the metric model used to estimate hospital migration in the Italian regions

We then grouped the categories to arrive at the three categories of interest, namely E-Environment, S-Social and G-Governance. Once this distribution was obtained, we further selected the variables through p-value analysis, choosing, through a strictly statistical and quantitative method, the variables that made the most sense for the model. Therefore, our method was based first on a classification carried out with a qualitative method, which led to the assignment of the variables and categories in the macro-items E, S and G. Then we applied a strictly quantitative method, i.e. the evaluation of the p -value, in determining the variables that made statistical sense for the purpose of the analysis (Fig. 1).

## Methodology

The methodology used for the analysis of the HEAR value in the context of ESG data is based on three complex tools, namely: econometric panel data models, clustering with the k-Means algorithm optimized with the Silhouette coefficient, machine-learning algorithms for prediction. Below we analyze in detail the methodological choices made for the analysis of hospital emigration in the Italian regions.

### Econometric panel data models

Panel data models have become an invaluable tool in econometrics, especially for analyzing phenomena that evolve both over time and across different entities. When examining hospital emigration in Italian regions, the utilization of panel data models presents numerous advantages and some notable limitations. Understanding these can provide a comprehensive view of their applicability and constraints, enhancing the analysis of such complex socio-economic phenomena [7, 28, 87].

Panel data models have advantages that we indicate below:

- Control of individual heterogeneity: panel data models excel in controlling for individual heterogeneity, which is critical when analyzing hospital emigration across different Italian regions. Each region possesses unique characteristics, such as distinct healthcare policies, economic conditions, and demographic profiles. Panel data models allow for the inclusion of these differences, ensuring more accurate and reliable estimates compared to models that use only cross-sectional or time-series data [19, 44, 104].
- Enhanced data informative: by combining cross-sectional and time-series data, panel data models provide more informative datasets. This increased variability and reduced collinearity among variables enhance the efficiency of econometric estimates. For example, tracking hospital emigration trends over multiple years across various regions can reveal patterns and correlations that might be overlooked in a pure cross-sectional analysis [11, 38, 39].
- Dynamic analysis capabilities: one of the significant strengths of panel data models is their ability to study dynamics and evolution over time. This is essential for understanding trends in hospital emigration, as these models can capture the impact of policy changes, economic shifts, and other time-varying factors on emigration rates. For instance, the effects of new healthcare regulations or economic crises on hospital emigration can be analyzed more precisely using panel data, providing deeper insights into temporal changes [3, 15, 18] .
- Reduction of bias: panel data models help mitigate the risk of omitted variable bias by accounting for unobserved individual heterogeneity. This is particularly important in healthcare studies where unobserved factors, such as regional healthcare quality or patient preferences, could significantly influence the results. By controlling for these factors, panel data models provide more robust and reliable findings [27, 67, 84].
- Complex behavioural modeling: the use of panel data allows for more sophisticated behavioural modeling, including interactions between time and entity effects. This capability can provide insights into how the same policy may have different impacts in different regions or how a region’s past emigration trends influence future patterns. For example, the responsiveness of hospital emigration to healthcare policy changes can vary significantly across regions, and panel data models can capture these nuanced behaviors [11, 17, 86].

However, the use of econometric models of the panel data type also presents limitations as indicated below:

- Challenges in data collection and quality: one of the primary limitations of panel data models is the complexity and cost associated with collecting panel data. For studying hospital emigration in Italian regions, it requires consistent and comprehensive data across multiple periods and regions. Inconsistencies or gaps in data can lead to biased estimates or limit the model’s applicability. Moreover, the accuracy and reliability of the data collected play a crucial role in the validity of the model’s results [37, 49, 58].
- Model complexity and estimation: panel data models can be complex to estimate and interpret, requiring sophisticated statistical techniques and a solid understanding of econometrics. This complexity can be a barrier for researchers or policymakers who do not have advanced econometric training, potentially limiting the accessibility and application of the findings. Understanding and applying the appropriate estimation techniques, such as fixed effects or random effects models, is crucial but can be challenging without specialized knowledge [11, 50, 56].
- Endogeneity issues: despite their ability to control for unobserved heterogeneity, panel data models are still susceptible to endogeneity issues, such as simultaneity and measurement errors. For instance, there might be feedback loops between hospital emigration and regional healthcare quality that are challenging to disentangle. These endogeneity issues can lead to biased and inconsistent estimates, undermining the reliability of the conclusions drawn from the model [13, 85, 96].
- Attrition and missing data: attrition, or the loss of data points over time, is a common problem in panel data studies. This can lead to incomplete datasets, which might bias the results if the attrition is non-random. In the context of Italian regions, regions with higher emigration might systematically have poorer data collection, skewing the results. Addressing attrition and handling missing data appropriately is essential to ensure the validity of the panel data model’s findings [65, 89, 105].
- Assumptions of stationarity and homogeneity: many panel data models assume that the relationships between variables are homogeneous across entities and stationary over time. However, these assumptions may not hold in reality. Different regions might respond differently to the same factors influencing hospital emigration, and these responses might change over time. For example, the impact of economic downturns on hospital emigration might vary significantly across regions, challenging the assumption of homogeneity [18, 26, 79].
- Computational intensity: estimating panel data models can be computationally intensive, especially with large datasets or complex models. This can limit the feasibility of using these models in some research settings, particularly where computational resources are constrained. The need for powerful computing capabilities and advanced software can pose a significant barrier to the widespread application of panel data models [61, 64, 100].
- Interpretation challenges: the results from panel data models can sometimes be difficult to interpret, particularly when there are multiple fixed or random effects. The inclusion of numerous control variables to account for unobserved heterogeneity can also complicate the interpretation of key coefficients. Clear and precise interpretation of the results is essential to ensure that the findings are useful and actionable for policymakers and stakeholders [11, 57, 75].

In conclusion, panel data models offer powerful advantages for analyzing hospital emigration in Italian regions by providing richer, more detailed data and controlling for unobserved heterogeneity. They enable dynamic analyses and reduce biases, leading to more reliable and nuanced insights. However, these benefits come with significant challenges, including data collection difficulties, model complexity, and potential issues with endogeneity and missing data. Researchers must carefully weigh these advantages and limitations when choosing panel data models for their analyses. The choice of model should be guided by the specific research questions, the nature of the data available, and the intended use of the findings. Despite the challenges, the strengths of panel data models make them a valuable tool for understanding complex socio-economic phenomena like hospital emigration in Italian regions.

### Clustering with the k-Means algorithm optimized with the Silhouette coefficient

The optimized k-Means clustering algorithm, enhanced with the Silhouette coefficient, presents a robust method for analyzing hospital emigration trends across Italian regions, bringing several advantages and limitations that are crucial for comprehensive understanding [66].

Using the k-Means algorithm has a set of advantages in the application as follows:

- Enhanced clustering accuracy: the optimized k-Means algorithm improves clustering accuracy by refining the initial centroid selection and optimizing the number of clusters. This is particularly beneficial for hospital emigration data, which can be influenced by a multitude of factors such as economic conditions, healthcare policies, and regional demographics. Accurate clustering helps in identifying true patterns and trends in the data, reducing the impact of outliers and noise [8, 25, 78].
- Effective cluster validation: the Silhouette coefficient provides a clear and interpretable metric for cluster validation, measuring how similar each data point is to its own cluster compared to other clusters. This ensures that the clusters formed are well-defined and meaningful, facilitating better interpretation of hospital emigration patterns. It helps in determining the optimal number of clusters, thereby avoiding overfitting or under fitting the model [66, 74, 81, 82].
- Dynamic and temporal analysis: the combination of optimized k-Means and Silhouette analysis allows for dynamic analysis of hospital emigration trends over time. By periodically recalculating clusters and their Silhouette scores, analysts can monitor changes and emerging patterns, offering valuable insights into the temporal dynamics of patient migration. This is particularly useful for policymakers who need to adapt strategies in response to evolving healthcare needs and migration trends [52, 53, 94].
- Identification of underlying patterns: clustering helps in uncovering underlying patterns in hospital emigration that may not be immediately apparent through other analytical methods. For example, it can reveal whether specific regions consistently lose patients to particular other regions, suggesting systemic issues in healthcare quality or accessibility that need to be addressed. These insights can guide targeted interventions and policy adjustments [4, 31, 53].
- Data-driven decision-making: the insights gained from this analytical approach support data-driven decision-making. Policymakers and healthcare administrators can use the clusters to allocate resources more effectively, target interventions, and tailor healthcare services to meet the specific needs of different regional populations. This targeted approach can improve patient retention and reduce unnecessary emigration [33, 36, 91].
- Scalability and computational efficiency: the k-Means algorithm, especially when optimized, is computationally efficient and scalable. This makes it suitable for analyzing large datasets, allowing for comprehensive examination of extensive patient records across multiple regions and time periods without significant performance issues. Its scalability ensures that it can handle the growing amount of healthcare data effectively [21, 54, 92].

However, there are also limitations in using the k-Means algorithm for clustering with the Silhouette coefficient, as follows:

- Sensitivity to initial conditions: despite optimization, k-Means clustering can still be sensitive to the initial placement of centroids. Poor initial choices can lead to suboptimal clusters, which may misrepresent the true structure of the data. This sensitivity necessitates multiple runs with different initial conditions to ensure robust clustering results [23, 102, 103].
- Determining the optimal number of clusters: while the Silhouette coefficient aids in determining the optimal number of clusters, it is not always definitive. There can be ambiguity in the interpretation of Silhouette scores, especially when the differences between potential numbers of clusters are small. This can lead to challenges in deciding the exact number of clusters that best represent the data [35, 107, 108].
- Interpretation challenges: the results from k-Means clustering, even when validated with the Silhouette coefficient, can be difficult to interpret, especially when dealing with high-dimensional data and multiple fixed or random effects. The inclusion of numerous control variables to account for unobserved heterogeneity can further complicate the interpretation of key clusters and their significance. Clear and precise interpretation of the results is essential to ensure that the findings are actionable and useful for policymakers [5, 29, 88].
- Handling complex relationships: k-Means clustering assumes that clusters are spherical and equally sized, which may not always be the case in real-world data. Hospital emigration patterns might exhibit more complex relationships that k-Means cannot capture effectively. This limitation can result in less accurate clustering, potentially overlooking nuanced migration behaviors and influencing factors [32, 45, 70].
- Computational intensity with large datasets: although k-Means is generally efficient, optimizing it for very large datasets can still be computationally intensive. This can limit its feasibility in some research settings where computational resources are constrained. The need for powerful computing capabilities and advanced software can pose a barrier to the widespread application of this method [21, 48, 92].
- Dependence on data quality: the effectiveness of k-Means clustering heavily depends on the quality and completeness of the data. Inconsistent or incomplete data can lead to biased estimates and inaccurate clusters, reducing the validity of the analysis. Ensuring high-quality data collection and pre-processing is critical to obtaining reliable and meaningful results [16, 24, 93].
- Static nature of the algorithm: while k-Means can be recalculated periodically, it does not inherently account for the dynamic nature of data. Real-time changes and sudden shifts in hospital emigration trends might not be captured promptly, potentially delaying necessary policy responses and adjustments [41, 95, 106].

In conclusion, the optimized k-Means clustering algorithm, enhanced with the Silhouette coefficient, offers significant advantages for analyzing hospital emigration in Italian regions. It improves clustering accuracy, facilitates effective cluster validation, supports dynamic analysis, and uncovers underlying patterns, all of which contribute to data-driven decision-making and better resource allocation. However, its sensitivity to initial conditions, interpretation challenges, computational demands, and dependence on data quality are notable limitations. Policymakers and researchers must carefully consider these advantages and limitations to maximize the utility of this method in understanding and addressing hospital emigration trends. By leveraging these analytical tools judiciously, they can make more informed decisions to enhance healthcare delivery and reduce unnecessary patient emigration across Italian regions.

### Machine learning algorithms for prediction

Machine learning (ML) algorithms are increasingly used for predictive tasks across various domains, offering numerous benefits as well as some limitations. This summary examines the advantages and disadvantages of using ML algorithms for prediction.

Below we consider the benefits of using machine learning algorithms for prediction.

- Enhanced accuracy: ML algorithms, such as Support Vector Machines (SVMs) and Neural Networks, can improve predictive accuracy significantly compared to traditional methods. For instance, ML improved cardiovascular risk prediction accuracy by exploiting complex interactions between risk factors, outperforming established algorithms [101].
- Handling complex data: ML techniques are particularly effective in analyzing large and complex datasets, uncovering patterns and relationships that might be missed by traditional statistical methods. This capability has been demonstrated in medical applications like disease forecasting and automated imaging analysis [1].
- Versatility across domains: ML algorithms are versatile and can be applied in various fields including finance, healthcare, education, and more. They have been used effectively for tasks such as stock market prediction, student performance forecasting, and heart disease detection [77, 98].
- Improvement with data: the performance of ML models often improves as more data becomes available, enabling more accurate and robust predictions over time [90].

Below we consider the limitations of using machine learning algorithms for prediction

- Data quality and quantity: the effectiveness of ML algorithms heavily depends on the quality and quantity of data. Poor quality data or insufficient data can lead to inaccurate predictions and biased outcomes [71].
- Complexity and interpretability: some ML models, particularly deep learning models, are often considered "black boxes" due to their complexity. This lack of interpretability can be a significant drawback, especially in fields where understanding the decision-making process is crucial [69].
- Computational resources: ML algorithms, especially those involving large neural networks, can be computationally intensive, requiring substantial processing power and memory. This can be a limitation in environments with limited computational resources [76].
- Bias and overfitting: ML models can suffer from overfitting, where they perform well on training data but poorly on new, unseen data. Additionally, they can propagate and even amplify biases present in the training data, leading to unfair or unethical outcomes [99].

Machine learning algorithms offer significant benefits in terms of accuracy, versatility, and the ability to handle complex data, but they also come with limitations related to data dependency, interpretability, computational demands, and potential for bias. Despite these challenges, the advantages of ML make it a powerful tool for predictive tasks across various domains.

## Econometric models for the estimation of the impact of the ESG determinants on the on hospital emigration at a regional level

Below we present an econometric analysis aimed at estimating ESG factors in determining hospital emigration in Italian regions. The analysed data were acquired by ISTAT-BES. The econometric techniques used are indicated below: Panel Data with Fixed Effects, Panel Data with Random Effects, Pooled Ordinary Least Squares-OLS, Weighted Least Squares-WLS, and Dynamic Panel at 1 Stage. To control for endogeneity we used the instrumental variable estimation applied to each of the components of the ESG model. The results are discussed critically.

### The estimation of the impact of the E-component within the ESG model on HEAR

Below we present the estimate of the value of the impact of the E-Component on HEAR. In particular, the following equation was estimated, namely:

$${\varvec{H}}{\varvec{E}}{\varvec{A}}{{\varvec{R}}}_{{\varvec{i}}{\varvec{t}}}={\boldsymbol{\alpha }}_{{\varvec{i}}}+{{\varvec{\beta}}}_{1}{\left({\varvec{D}}{\varvec{L}}{\varvec{P}}\right)}_{{\varvec{i}}{\varvec{t}}}+{{\varvec{\beta}}}_{2}{\left({\varvec{C}}{\varvec{L}}{\varvec{D}}\right)}_{{\varvec{i}}{\varvec{t}}}+{{\varvec{\beta}}}_{3}{\left({\varvec{D}}{\varvec{I}}{\varvec{H}}{\varvec{P}}\right)}_{{\varvec{i}}{\varvec{t}}}+{{\varvec{\beta}}}_{4}{\left({\varvec{D}}{\varvec{W}}{\varvec{I}}{\varvec{P}}\right)}_{{\varvec{i}}{\varvec{t}}}+{{\varvec{\beta}}}_{5}{\left({\varvec{P}}{\varvec{A}}\right)}_{{\varvec{i}}{\varvec{t}}}+{{\varvec{\beta}}}_{6}{\left({\varvec{S}}{\varvec{S}}{\varvec{C}}\right)}_{{\varvec{i}}{\varvec{t}}}+{{\varvec{\beta}}}_{7}{\left({\varvec{A}}{\varvec{U}}{\varvec{G}}\right)}_{{\varvec{i}}{\varvec{t}}}+{{\varvec{\beta}}}_{8}{\left({\varvec{T}}{\varvec{M}}{\varvec{W}}\right)}_{{\varvec{i}}{\varvec{t}}}$$

Where $$\text{i}=20$$ and $$\text{t}=[2004;2021]$$.

The equation was estimated through the use of the following econometric techniques: Pooled OLS, Panel Data with Fixed Effects, Panel Data with Random Effects, Weighted Least Squares-WLS and 1-Step Dynamic Panel. The variables analyzed in the model are reported in Table 5.

**Table 5:** Definition of Variables used for the Estimation of the Impact of E-Environmental on ESG. Source: ISTAT-BES

Variables	Label	Definition
$y$	HEAR	Percentage ratio between hospital discharges in regions other than that of residence and the total of the resignations of residents in the region. Data yes refer only to hospital admissions under the ordinary "acute" regime (admissions to wards are excluded of “spinal unit”, “functional recovery and rehabilitation”, “neuro-rehabilitation” and “long-term care”).
${x}_{1}$	DLP	Percentage of people aged 14 and over who declare that the landscape of the place they live is affected by evident degradation out of the total number of people aged 14 and over.
${x}_{2}$	CLD	Percentage of people aged 14 and over who list landscape damage caused by excessive building construction as one of the five most worrying environmental problems among all people aged 14 and over.
${x}_{3}$	DIHP	Number of days in the year in which the maximum temperature is above the 90th percentile of the distribution in the reference climatological period (1981-2010), for at least six consecutive days.
${x}_{4}$	DWIP	Number of days of the year in which the daily cumulative precipitation exceeds or equals the value of 50 mm
${x}_{5}$	PA	Percentage of land surface covered by terrestrial protected natural areas included in the official list of protected areas (Euap) or belonging to the Natura 2000 network.
${x}_{6}$	SSC	Percentage of authorized bathing coasts out of the total coastal line in accordance with current regulations.
${x}_{7}$	AUG	Square meters of urban greenery per inhabitant in provincial capitals/metropolitan cities
${x}_{8}$	TMW	Percentage of municipal waste sent to landfill out of your total municipal waste produced

We find that the level of HEAR is positively associated to:

- *DLP:* it is the percentage of people aged 14 and over who declare that the landscape of the place where they live is affected by evident degradation out of the total of people aged 14 and over. There is a positive relationship between the value of HEAR and the value of DLP. Regions in which landscape conditions are worse tend to be characterized by greater hospital emigration.
- *DWIP:* represents the number of days of the year in which the daily cumulative precipitation exceeds or equals the value of 50 mm. There is a positive relationship between the value of HEAR and the value of DWIP. Regions with high levels of daily precipitation also have higher levels of hospital emigration.
- *PA:* is the percentage of the earth's surface covered by terrestrial natural protected areas included in the official list of protected areas or belonging to the Natura 2000 network. The regions in which there is a growth in protected areas tend to also be the regions in which there is it is an increase in hospital emigration. It should be considered that the regions that have the greatest hospital emigration are the Italian regions with low populations, where a significant part of the territory appears to be devoid of urbanisation.
- *SSC:* represents the percentage of authorized bathing coasts on the total coastline according to current legislation. There is a positive relationship between the value of the percentage of bathing coasts and the value of hospital emigration. The regions that have a greater supply of bathing coasts also have a greater supply of hospital migration.
- *AUG:* represents the value of square meters of urban greenery per inhabitant in provincial capitals/metropolitan cities. There is a positive relationship between the value of square meters of urban greenery per inhabitant and the value of hospital emigration. The value of hospital emigration tends to grow with the urban greenery detected in metropolitan areas.
- *TMW:* is a variable that considers the percentage of municipal waste sent to landfill compared to the total municipal waste produced. There is a positive relationship between the value of TMW and the value of HEAR. The regions in which the value of municipal waste in landfill tends to increase are also regions in which the value of hospital emigration tends to increase.

We find that the level of HEAR is negatively associated to:

- *CLD:* is a variable that considers the percentage of people 14 years and older who list landscape damage caused by excessive building construction as one of the five most concerning environmental problems among all people 14 years and older. There is a negative relationship between the CLD value and the HEAR value. Regions that have a higher level of concern about landscape deterioration tend to have lower hospital emigration.
- *DIHP:* is the number of days in the year in which the maximum temperature is above the 90th percentile of the distribution in the reference climatological period (1981-2010), for at least six consecutive days. There is a positive relationship between the DIHP value and the HEAR value. Regions that have high levels of DIHP also have high levels of HEAR.

The estimated econometric results have been summarized in the following Table 6.

**Table 6:** Estimation of the impact of a set of E-Environmental Variables on HEAR in the Italian Regions. Data from ISTAT-BES

	Label	Costant	DLP	CLD	DIHP	DWIP	PA	SSC	AUG	TMW	HEAR(-1)
Pooled OLS	Coefficient	416.7	0.15	-0.23	-0.07	0.97	0.11	0.03	0.02	0.10	
	Standard Error	0.86	0.03	0.06	0.03	0.36	0.03	0.01	0.00	0.01	
	P-Value	***	***	***	**	***	***	***	***	***	
Fixed Effetcs	Coefficient	670.64	0.17	-0.14	-0.15	0.81	0.09	0.02	0.01	0.06	
	Standard Error	0.64	0.02	0.03	0.01	0.18	0.013	0.00	0.00	0.01	
	P-Value	***	***	***	***	***	***	***	***	***	
Random Effects	Coefficient	659.71	0.17	-0.15	-0.15	0.80	0.09	0.02	0.01	0.06	
	Standard Error	126.33	0.02	0.03	0.01	0.18	0.01	0.00	0.00	0.01	
	P-Value	***	***	***	***	***	***	***	***	***	
WLS	Coefficient	224.66	0.17	-0.11	-0.06	0.60	0.08	0.02	0.02	0.12	
	Standard Error	0.34	0.02	0.03	0.01	0.16	0.01	0.00	0.00	0.00	
	P-Value	***	***	***	***	***	***	***	***	***	
1-step dynamic panel	Coefficient		0.15	-0.12	-0.13	0.68	0.08	0.01	0.02	0.09	0.20
	Standard Error		0.02	0.02	0.02	0.20	0.01	0.00	0.00	0.01	0.23
	P-Value		***	***	***	***	***	***	***	***	

To estimate the overall value of the E-component on HEAR we calculated the average of each variable considering the values obtained respectively through the five econometric models analyzed.

#### Instrumental variable model

To verify the presence of endogeneity in the analysis carried out, we chose to apply the instrumental variables method. The instrumental variables used refer to the innovation, research and creativity grouping of the ISTAT-BES database. In particular, the estimated equation is indicated below:

$${\varvec{H}}{\varvec{E}}{\varvec{A}}{{\varvec{R}}}_{{\varvec{i}}{\varvec{t}}}={\boldsymbol{\alpha }}_{{\varvec{i}}}+{{\varvec{\beta}}}_{1}{\left({\varvec{D}}{\varvec{L}}{\varvec{P}}\right)}_{{\varvec{i}}{\varvec{t}}}+{{\varvec{\beta}}}_{2}{\left({\varvec{C}}{\varvec{L}}{\varvec{D}}\right)}_{{\varvec{i}}{\varvec{t}}}+{{\varvec{\beta}}}_{3}{\left({\varvec{S}}{\varvec{S}}{\varvec{C}}\right)}_{{\varvec{i}}{\varvec{t}}}+{{\varvec{\beta}}}_{4}{\left({\varvec{A}}{\varvec{U}}{\varvec{G}}\right)}_{{\varvec{i}}{\varvec{t}}}+{{\varvec{\beta}}}_{5}{\left({\varvec{P}}{\varvec{P}}{\varvec{T}}\right)}_{{\varvec{i}}{\varvec{t}}}+{{\varvec{\beta}}}_{6}{\left({\varvec{I}}{\varvec{P}}{\varvec{S}}\right)}_{{\varvec{i}}{\varvec{t}}}+{{\varvec{\beta}}}_{7}{\left({\varvec{K}}{\varvec{W}}\right)}_{{\varvec{i}}{\varvec{t}}}+{{\varvec{\beta}}}_{8}{\left({\varvec{C}}{\varvec{C}}{\varvec{E}}\right)}_{{\varvec{i}}{\varvec{t}}}+{{\varvec{\beta}}}_{9}{\left({\varvec{M}}{\varvec{I}}{\varvec{G}}\right)}_{{\varvec{i}}{\varvec{t}}}+{{\varvec{\beta}}}_{10}{\left({\varvec{R}}{\varvec{I}}{\varvec{U}}\right)}_{{\varvec{i}}{\varvec{t}}}+{{\varvec{\beta}}}_{11}{\left({\varvec{O}}{\varvec{N}}{\varvec{E}}{\varvec{P}}{\varvec{C}}\right)}_{{\varvec{i}}{\varvec{t}}}+{{\varvec{\beta}}}_{12}{\left({\varvec{M}}4{\varvec{F}}{\varvec{A}}{\varvec{M}}\right)}_{{\varvec{i}}{\varvec{t}}}+{{\varvec{\beta}}}_{13}{\left({\varvec{C}}10{\varvec{E}}\right)}_{{\varvec{i}}{\varvec{t}}}$$

Where $\text{i}=20$ and $\text{t}=[2004;2021]$, where DLP, CLD and SSC are instrumented variable-X and PPT, IPS, KW, CCE, MIG, RIU, ONEPC, M4FAM, C10E are instrumental variables-Z. The variables used are indicated in the following Table 7:

**Table 7:** Instrumental Variable Estimation

IV Model	Variables	Acronym	Coefficient	Standard Error	P-value
X-Instrumented Variables	Dissatisfaction with the landscape of the living place	DLP	9.98	0.75	***
	Concern about landscape deterioration	CLD	0.29	0.08	***
	Swimming sea coasts	SSC	-1.17	0.17	***
	Availability of urban greenery	AUG	0.9	0.02	***
Z-Instrumental Variables	Propensity towards patenting	PPT	Statistics		
	Innovation of the production system	IPS	Mean Dependent Variable		10.1
	Knowledge workers	KW	Sum Squared Residual		211157.64
	Cultural and creative employment	CCE	R-Squared		0.2
	Mobility of Italian graduates (25-39 years)	MIG	F(4,335)		12.53
	Regular internet users	RIU	S.D. Dependent Variable		6.4
	Availability of at least one computer and Internet connection in the family	ONEPC	S.E. of Regression		7.94
	Municipalities with entirely online services for families	M4FAM	Adjusted R-Squared		0.25
	Companies with at least 10 employees with web sales to end customers	C10E	P-Value (F)		1.62e-09

Therefore it appears that the HEAR level is positively associated with DLP, CLD and AUG, while it is negatively associated with SSC. The data suggests that hospital migration tends to increase as environmental conditions deteriorate. Regions characterized by greater environmental degradation present higher levels of hospital migration.

### The Estimation of the S-Social Component within the ESG Model on the Value of HEAR

Below we analyze the impact of a set of variables relating to the S-Social component of the ESG model on the value of hospital emigration. The analyzed data refers to the ISTAT-BES database. The econometric techniques used are indicated below: Panel Data with Fixed Effects, Panel Data with Random Effects, Pooled OLS, Weighted Least Squares-WLS. Specifically, the following equation was estimated:

$${\varvec{H}}{\varvec{E}}{\varvec{A}}{{\varvec{R}}}_{{\varvec{i}}{\varvec{t}}}={\boldsymbol{\alpha }}_{{\varvec{i}}}+{{\varvec{\beta}}}_{1}{\left({\varvec{T}}{\varvec{U}}\right)}_{{\varvec{i}}{\varvec{t}}}+{{\varvec{\beta}}}_{2}{\left({\varvec{E}}{\varvec{X}}\right)}_{{\varvec{i}}{\varvec{t}}}+{{\varvec{\beta}}}_{3}{\left({\varvec{E}}{\varvec{R}}\right)}_{{\varvec{i}}{\varvec{t}}}+{{\varvec{\beta}}}_{4}{\left({\varvec{L}}{\varvec{P}}{\varvec{E}}\right)}_{{\varvec{i}}{\varvec{t}}}+{{\varvec{\beta}}}_{5}{\left({\varvec{R}}{\varvec{I}}{\varvec{P}}{\varvec{D}}\right)}_{{\varvec{i}}{\varvec{t}}}+{{\varvec{\beta}}}_{6}{\left({\varvec{R}}{\varvec{O}}{\varvec{P}}\right)}_{{\varvec{i}}{\varvec{t}}}+{{\varvec{\beta}}}_{7}{\left({\varvec{E}}{\varvec{P}}{\varvec{I}}{\varvec{H}}{\varvec{C}}\right)}_{{\varvec{i}}{\varvec{t}}}+{{\varvec{\beta}}}_{8}{\left({\varvec{G}}{\varvec{P}}{\varvec{T}}\right)}_{{\varvec{i}}{\varvec{t}}}+{{\varvec{\beta}}}_{9}{\left({\varvec{D}}{\varvec{R}}{\varvec{s}}\right)}_{{\varvec{i}}{\varvec{t}}}$$

Where $i=20$ and $t=[2004;2021]$. The list of variables used in the model are showed in Table 8.

**Table 8:** Definition of Variables used for the Estimation of the Impact of E-Environmental on HEAR

Variables	Label	Definition
$y$	HEAR	Percentage ratio between hospital discharges in regions other than that of residence and the total of the resignations of residents in the region. Data yes refer only to hospital admissions under the ordinary "acute" regime (admissions to wards are excluded of “spinal unit”, “functional recovery and rehabilitation”, “neuro-rehabilitation” and “long-term care”).
${x}_{1}$	TU	Percentage of recent high school graduates who enrol at university for the first time in the same year in which they obtained their upper secondary school diploma (cohort specific rate). Those enrolled in Higher Technical Institutes, Institutes of Higher Artistic, Musical and Dance Education, Higher Schools for Linguistic Mediators and foreign universities are excluded.
${x}_{2}$	EX	Percentage of people aged 18-24 with at most a lower secondary school diploma (middle school diploma), who do not possess regional professional qualifications obtained in courses lasting at least 2 years and not included in an education or training course out of the total number of people aged 18-24.
${x}_{3}$	ER	Percentage of employed people aged 20-64 in the population aged 20-64.
${x}_{4}$	LPE	Percentage of employees with an hourly wage lower than 2/3 of the median wage out of total employees.
${x}_{5}$	RIPD	Number of fatal accidents and those resulting in permanent disability among the total employed (net of the armed forces) per 10,000.
${x}_{6}$	ROP	Percentage of people living in families with an equivalent net income below a poverty risk threshold, set at 60% of the median of the individual distribution of equivalent net income. The income reference year is the calendar year preceding the survey year.
${x}_{7}$	EPIHC	Percentage of elderly people treated in integrated home care out of the total resident elderly population (65 years and over).
${x}_{8}$	GPT	Percentage of general practitioners with a number of patients exceeding the maximum threshold of 1500 patients envisaged by the contract for general practitioners.
${x}_{9}$	DRs	Number of doctors per 1,000 inhabitants.

We found that the level of HEAR is positively associated to:

- *TU:* is the percentage of recent high school graduates who enrol at university for the first time in the same year in which they obtained their high school diploma (cohort-specific rate). There is a positive relationship between the value of TU and the value of HEAR. Regions that have a high level of recent high school graduates also have higher levels of HEAR.
- *LPE:* Percentage of employees with an hourly wage lower than 2/3 of the median wage out of total employees. There is a positive relationship between the value of low-paid employees and the value of HEAR. Regions that have a high number of low-paid employees also have a high level of hospital emigration.
- *RIPD:* Number of fatal accidents and those with permanent disability among the total employed (net of the armed forces) per 10,000. There is a positive relationship between the number of fatal accidents and those resulting in permanent disability and the HEAR value in the Italian regions. Specifically, it is possible to note that regions that have higher levels of RIPD also have high levels of HEAR.
- *ROP:* is the percentage of people living in families with an equivalent net income below the poverty risk threshold, set at 60% of the median of the individual distribution of equivalent net income. The income reference year is the calendar year preceding the survey year. There is a positive relationship between the ROP value and the HEAR value. Regions that have high ROP values also have high HEAR values.
- *EPIHC:* is the percentage of elderly people treated in integrated home care out of the total resident elderly population (65 years and over). There is a positive relationship between the EPIHC value and the HEAR value. Regions where there are more elderly people treated in integrated home care have higher levels of hospital migration.

We also found that the level of HEAR is negatively associated with:

- *EX:* is the percentage of people aged 18-24 with a maximum of a lower secondary school diploma (middle school diploma), who do not possess regional professional qualifications obtained in courses lasting at least 2 years and not included in an education or training course out of the total people aged between 18 and 24. There is a negative relationship between the value of EX and the value of HEAR. Regions where early exit from the school system is lower have higher levels of HEAR value.
- *ER:* is the percentage of employed people aged between 20 and 64 in the population aged 20-64. There is a negative relationship between the value of employed people and the value of hospital emigration. Regions where the value of hospital emigration tends to increase tend to have a reduced level of employment.
- *GPT:* is the percentage of general practitioners with a number of patients exceeding the maximum threshold of 1500 patients envisaged by the contract for general practitioners. There is a negative relationship between the GPT value and the HEAR value. The regions where the number of doctors with a maximum threshold of 1500 assisted decreases are associated with a growth in the value of HEAR.
- *DRs:* it represents the number of doctors per 1,000 inhabitants. There is a negative relationship between the value of DRs and the value of HEAR. The regions where the number of doctors decreases are characterized by an increasing value of hospital emigration.

The econometric estimations are showed in Table 9.

**Table 9:** Estimation of the impact of a set of S-Social Variables on HEAR in the Italian Regions

		Constant	TU	EX	ER	LPE	RIPD	ROP	EPIHC	GPT	DRs
Fixed-effects	Coefficient	707.280	0.034	-0.073	-0.111	0.040	0.308	0.184	0.460	-0.020	-0.435
	Standard Error	0,676	0.006	0.031	0.014	0.017	0.039	0.036	0.087	0.011	0.087
	P-value	***	***	**	***	**	***	***	***	*	***
Pooled OLS	Coefficient	116.305	0.062	-0.386	-0.112	0.102	0.480	0.073	0.560	-0.164	-0.706
	Standard Error	0,938	0.020	0.105	0.049	0.059	0.131	0.036	0.288	0.025	0.287
	P-value	***	***	***	**	*	***	**	*	***	**
Random-effects	Coefficient	714.110	0.034	-0.075	-0.111	0.040	0.309	0.180	0.461	-0.021	-0.435
	Standard Error	144.201	0.006	0.031	0.014	0.017	0.039	0.035	0.087	0.011	0.087
	P-value	***	***	**	***	**	***	***	***	*	***
WLS	Coefficient	104.708	0.052	-0.289	-0.098	0.152	0.377	0.059	0.573	-0.139	-0.583
	Standard Error	0,590	0.013	0.095	0.037	0.042	0.094	0.028	0.202	0.017	0.195
	P-value	***	***	***	***	***	***	**	***	***	***

#### Instrumental variables

To check for the presence of endogeneity we created the following model with instrumental variables. The instrumental variables were acquired from the ISTAT-BES database using the political and institutions macro-category. Specifically we estimated the following equations:

$${\varvec{H}}{\varvec{E}}{\varvec{A}}{{\varvec{R}}}_{{\varvec{i}}{\varvec{t}}}={\boldsymbol{\alpha }}_{{\varvec{i}}}+{{\varvec{\beta}}}_{1}{\left({\varvec{T}}{\varvec{U}}\right)}_{{\varvec{i}}{\varvec{t}}}+{{\varvec{\beta}}}_{2}{\left({\varvec{E}}{\varvec{R}}\right)}_{{\varvec{i}}{\varvec{t}}}+{{\varvec{\beta}}}_{3}{\left({\varvec{R}}{\varvec{I}}{\varvec{P}}{\varvec{D}}\right)}_{{\varvec{i}}{\varvec{t}}}+{{\varvec{\beta}}}_{4}{\left({\varvec{E}}{\varvec{P}}{\varvec{I}}{\varvec{H}}{\varvec{C}}\right)}_{{\varvec{i}}{\varvec{t}}}+{{\varvec{\beta}}}_{5}{\left({\varvec{D}}{\varvec{R}}{\varvec{s}}\right)}_{{\varvec{i}}{\varvec{t}}}+{{\varvec{\beta}}}_{6}{\left({\varvec{E}}{\varvec{P}}\right)}_{{\varvec{i}}{\varvec{t}}}+{{\varvec{\beta}}}_{7}{\left({\varvec{T}}{\varvec{I}}{\varvec{P}}\right)}_{{\varvec{i}}{\varvec{t}}\boldsymbol{ }}+{{\varvec{\beta}}}_{8}{\left({\varvec{T}}{\varvec{I}}{\varvec{J}}{\varvec{S}}\right)}_{{\varvec{i}}{\varvec{t}}}+{{\varvec{\beta}}}_{9}{\left({\varvec{T}}{\varvec{P}}\right)}_{{\varvec{i}}{\varvec{t}}}+{{\varvec{\beta}}}_{10}{\left({\varvec{T}}{\varvec{P}}{\varvec{F}}\right)}_{{\varvec{i}}{\varvec{t}}}+{{\varvec{\beta}}}_{11}{\left({\varvec{W}}{\varvec{R}}{\varvec{P}}\right)}_{{\varvec{i}}{\varvec{t}}}+{{\varvec{\beta}}}_{12}{\left({\varvec{W}}{\varvec{P}}{\varvec{R}}{\varvec{L}}{\varvec{L}}\right)}_{{\varvec{i}}{\varvec{t}}}+{{\varvec{\beta}}}_{13}{\left({\varvec{A}}{\varvec{A}}{\varvec{I}}{\varvec{P}}\right)}_{{\varvec{i}}{\varvec{t}}}+{{\varvec{\beta}}}_{14}{\left({\varvec{D}}{\varvec{C}}{\varvec{P}}\right)}_{{\varvec{i}}{\varvec{t}}}+{{\varvec{\beta}}}_{15}{\left({\varvec{C}}{\varvec{P}}\right)}_{{\varvec{i}}{\varvec{t}}}$$

where $i=20$ and $t=[2004;2021]$. The list of variables used in the model are showed in Table 4. The data demonstrate that the value of HEAR is positively associated with DRs and negatively associated with TU, ER, RIPD, EPIHC (Table 10).

**Table 10:** Instrumental Variable Estimation

		Acronym	Coefficient	Standard Error	p-Value
Categories	Variables	const	10.41	0.63	***
X	Transition to university	TU	-0.17	0.08	**
	Employment rate (20-64 years)	ER	-0.47	0.13	***
	Rate of Injuries and Permanent Disability	RIPD	1.34	0.33	***
	Elderly people treated in integrated home care	EPIHC	-3.57	1.45	**
	Doctors	DRs	2.81	1.32	**
Z	Electoral participation	EP	Statistics		
	Trust in the Italian Parliament	TIP	Mean Dependent Variable		10.39
	Trust in the justice system	TIJS	Sum Squared Resid		22038.7
	Trust in parties	TP	R-Squared		0.00
	Trust in the police and firefighters	TPF	F(5,345)		3.85
	Women and political representation in Parliament	WPRP	S.D. Dependent variable		5.85
	Women and political representation at local level	WPRLL	S.E. of Regression		7.99
	Average age of Italian parliamentarians	AAIP	Adjusted R-Squared		0.00
	Duration of civil proceedings	DCP	P-Value		0.00
	Crowding in prisons	CP			

Overall we can see that hospital migration tends to grow with the reduction of the S-Social component of the ESG model. Specifically, we can note that the level of hospital migration tends to grow with the reduction of transition to university, with employment rate, and with the value of elderly people treated in integrated home care. The only variable of the S-Social component positively correlated with hospital migration is represented by the number of doctors at regional level. However, in a broad sense it is possible to state that the level of hospital migration tends to grow with the deterioration of the social condition.

### The estimation of the G-Governance component within the ESG model on the value of HEAR

Below we analyse the value of the impact of the variables relating to the G-Governance component within the ESG model on the value of the HEAR variable. The data used were acquired by ISTAT-BES. The following econometric models were applied namely: Panel Data with Fixed Effects, Panel Data with Random Effects, Pooled OLS, 1-Step Dynamic Panel. In particular we estimated the following equation:

$${\varvec{H}}{\varvec{E}}{\varvec{A}}{{\varvec{R}}}_{{\varvec{i}}{\varvec{t}}}={\boldsymbol{\alpha }}_{{\varvec{i}}{\varvec{t}}}+{{\varvec{\beta}}}_{1}{\left({\varvec{P}}{\varvec{Y}}{\varvec{C}}{\varvec{K}}\right)}_{{\varvec{i}}{\varvec{t}}}+{{\varvec{\beta}}}_{2}{\left({\varvec{P}}{\varvec{D}}{\varvec{A}}{\varvec{L}}\right)}_{{\varvec{i}}{\varvec{t}}}+{{\varvec{\beta}}}_{3}{\left({\varvec{P}}{\varvec{Y}}{\varvec{C}}{\varvec{C}}\right)}_{{\varvec{i}}{\varvec{t}}}+{{\varvec{\beta}}}_{4}{\left({\varvec{D}}{\varvec{C}}{\varvec{P}}\right)}_{{\varvec{i}}{\varvec{t}}}+{{\varvec{\beta}}}_{5}{\left({\varvec{R}}{\varvec{I}}{\varvec{U}}\right)}_{{\varvec{i}}{\varvec{t}}}+{{\varvec{\beta}}}_{6}{\left({\varvec{A}}{\varvec{A}}{\varvec{I}}{\varvec{P}}\right)}_{{\varvec{i}}{\varvec{t}}}+{{\varvec{\beta}}}_{7}{\left({\varvec{M}}{\varvec{I}}{\varvec{G}}\right)}_{{\varvec{i}}{\varvec{t}}\boldsymbol{ }}+{{\varvec{\beta}}}_{8}{\left({\varvec{C}}{\varvec{W}}10\right)}_{{\varvec{i}}{\varvec{t}}}$$

Where $i=20$ and $t=[2004;2021]$. A list of variables is showed in Table 11.

**Table 11:** The Variables Used for the Estimation of the Impact of G-Governance Component within the ESG model on HEAR

Variable	Label	Definition
$y$	HEAR	Percentage ratio between hospital discharges in regions other than that of residence and the total of the resignations of residents in the region. Data yes refer only to hospital admissions under the ordinary "acute" regime (admissions to wards are excluded of “spinal unit”, “functional recovery and rehabilitation”, “neuro-rehabilitation” and “long-term care”).
${x}_{1}$	PYCC	Percentage of people aged 14 and over who have non-cohabiting relatives (in addition to parents, children, brothers, sisters, grandparents, grandchildren), friends or neighbours to rely on out of the total number of people aged 14 and over.
${x}_{2}$	AAIP	Average age of parliamentarians in the Senate and the House. Senators and deputies elected in foreign constituencies and senators for life are excluded.
${x}_{3}$	DCP	Actual average duration in days of proceedings settled in ordinary courts.
${x}_{4}$	PYCK	Victims of pickpocketing per 1,000 inhabitants. The number of victims is calculated using data on victims who reported pickpocketing to the police, corrected with the number of victims who did not report taken from the Citizen Security Survey, through a specific correction factor for geographical distribution and a by sex and age group.
${x}_{5}$	PDAL	Presence of elements of degradation in the area where you live: Percentage of people aged 14 and more than that they often see elements of social degradation and environmental in the area in which they live (they often see at least one element of degradation among the following: people who take drugs, people who deal drugs, acts of vandalism against public property, prostitutes looking for clients) out of the total number of people 14 years and older.
${x}_{6}$	MIG	Migration rate of Italians (25-39 years) with qualifications of tertiary study, calculated as the ratio between the migratory balance (difference between registered and canceled per transfer of residence) and residents with title of tertiary study (undergraduate, AFAM, doctorate). Values for Italy they only include movements to/from abroad, for the divisional values the inter-departmental movements.
${x}_{7}$	RIU	Percentage of people aged 11 and over who used the Internet at least once a week in the 3 months preceding the interview.
${x}_{8}$	CW10	Percentage of companies with at least 10 employees who sold via the web to end customers (B2C) during the previous year. From the survey year 2021, economic activities from division 10 to 82 are considered based on the new Ateco 2007 classification (excluding the K-Financial and insurance activities section). From the same year of survey, the unit of analysis for which the estimates are provided is the enterprise, i.e. a statistical unit that can be made up of one or more legal units

Results show that the level of HEAR is positively associated to:

- *PYCK:* represents the number of pickpocketing victims per 1,000 inhabitants. The number of victims is calculated using the data of the victims who reported the pickpocketing to the police, corrected with the number of victims who did not report, obtained from the Citizen Security Survey, through a specific corrective factor for the distribution geographical and by sex and age groups. There is a positive relationship between the value of PYCK and the value of HEAR. The regions that have a higher prevalence of pickpocketing are also the regions that have a higher hospital emigration value.
- *PDAL:* represents the presence of elements of degradation in the area where you live: Percentage of people aged 14 and over who often see elements of social and environmental degradation in the area where they live (they often see at least one element of degradation among the following (people who use drugs, people who deal drugs, acts of vandalism against public property, prostitutes looking for clients) out of the total number of people aged 14 and over. There is a positive relationship between the value of degradation and the value of HEAR. The regions which have a higher level of degradation also have a higher level of hospital emigration.
- *PYCC:* is the percentage of people aged 14 and over who have non-cohabiting relatives (in addition to parents, children, brothers, sisters, grandparents, grandchildren), friends or neighbours to rely on out of the total number of people aged 14 and over. There is a positive relationship between the value of PYCC and the value of HEAR. Regions where the level of people to rely on tends to grow also have a higher level of hospital emigration.
- *DCP:*is the average effective duration in days of proceedings resolved before ordinary courts. There is a positive relationship between the value of DCP and the value of HEAR at the regional level. The regions in which the length of judicial proceedings increases are also the regions characterized by a high level of HEAR.

Results also show that the level of HEAR is negatively associated to:

- *RIU:* represents the Percentage of people aged 11 and over who used the Internet at least once a week in the 3 months before the interview. There is a negative relationship between the value of RIU and the value of HEAR. The regions in which the population uses the internet less frequently are also the regions with the greatest hospital emigration.
- *AAIP:*is the average age of parliamentarians in the Senate and the House. Senators and deputies elected in foreign constituencies and senators for life are excluded. Regions that have a lower level of AAP also have a higher level of HEAR. That is, as the age of deputies and senators increases, the value of hospital emigration decreases.
- *MIG:* is the migration rate of Italians (25-39 years) with qualifications of tertiary study, calculated as the ratio between the migratory balance (difference between registered and canceled for transfer of residence) and residents with title of tertiary study (undergraduate, AFAM, doctorate). Values for Italy they only include movements to/from abroad, for the divisional values the inter-departmental movements. There is a negative relationship between the mobility of Italian graduates and the value of hospital emigration. In fact, the regions in which the mobility of Italian graduates decreases tends to increase hospital emigration.
- *CW10:* represents the amount of companies with at least 10 employees with web sales to end customers. There is a negative relationship between the CW10 value and the HEAR value. In regions where the number of companies with at least 10 employees with web sales decreases, the value of hospital emigration increases.

The results of the econometric estimations are showed in Table 12.

**Table 12:** Estimation of the impact of a set of G-Governance Variables on HEAR in the Italian Regions

		const	PYCC	AAIP	DCP	PYCK	PDAL	MIG	RIU	CW10	HEAR
Fixed Effects	Coefficient	9.517	0.020	-0.075	0.002	0.318	0.118	-0.078	-0.029	-0.164	
	Standard Error	0.646	0.006	0.008	0.001	0.104	0.041	0.020	0.014	0.042	
	*P*-Value	***	***	***	***	***	***	***	**	***	
Pooled OLS	Coefficient	14.087	0.030	-0.051	0.005	-0.524	-0.153	-0.151	-0.046	-0.272	
	Standard Error	0.928	0.012	0.018	0.001	0.096	0.076	0.041	0.027	0.078	
	*P*-Value	***	**	***	***	***	**	***	*	***	
Random-effects	Coefficient	9.793	0.020	-0.074	0.003	0.263	0.111	-0.080	-0.030	-0.170	
	Standard Error	1.541	0.006	0.008	0.001	0.101	0.041	0.020	0.014	0.042	
	*P*-Value	***	***	***	***	***	***	***	**	***	
1-step dynamic panel	Coefficient		0.019	-0.074	0.003	0.339	0.144	-0.090	-0.075	-0.084	0.042
	Standard Error		0.004	0.008	0.001	0.090	0.036	0.026	0.018	0.043	0.282
	*P*-Value		***	***	**	***	***	***	***	**	

#### Instrumental variable models

In order to control for endogeneity we apply the instrumental variable model. The instrumental variables for controlling endogeneity are acquired from the landscape and cultural heritage category present within the ISTAT-BES database. In particular, we estimated the following equation:

$${\varvec{H}}{\varvec{E}}{\varvec{A}}{{\varvec{R}}}_{{\varvec{i}}{\varvec{t}}}={\boldsymbol{\alpha }}_{{\varvec{i}}{\varvec{t}}}+{{\varvec{\beta}}}_{1}{\left({\varvec{D}}{\varvec{C}}{\varvec{P}}\right)}_{{\varvec{i}}{\varvec{t}}}+{{\varvec{\beta}}}_{2}{\left({\varvec{R}}{\varvec{I}}{\varvec{U}}\right)}_{{\varvec{i}}{\varvec{t}}}+{{\varvec{\beta}}}_{3}{\left({\varvec{C}}{\varvec{W}}10\right)}_{{\varvec{i}}{\varvec{t}}}+{{\varvec{\beta}}}_{4}{\left({\varvec{M}}{\varvec{C}}{\varvec{E}}{\varvec{C}}\right)}_{{\varvec{i}}{\varvec{t}}}+{{\varvec{\beta}}}_{5}{\left({\varvec{D}}{\varvec{R}}{\varvec{M}}{\varvec{H}}\right)}_{{\varvec{i}}{\varvec{t}}}+{{\varvec{\beta}}}_{6}{\left({\varvec{I}}{\varvec{B}}\right)}_{{\varvec{i}}{\varvec{t}}}+{{\varvec{\beta}}}_{7}{\left({\varvec{E}}{\varvec{R}}{\varvec{S}}{\varvec{U}}{\varvec{D}}\right)}_{{\varvec{i}}{\varvec{t}}}+{{\varvec{\beta}}}_{8}{\left({\varvec{E}}{\varvec{U}}{\varvec{S}}{\varvec{D}}{\varvec{A}}\right)}_{{\varvec{i}}{\varvec{t}}}+{{\varvec{\beta}}}_{9}{\left({\varvec{P}}{\varvec{M}}{\varvec{A}}\right)}_{{\varvec{i}}{\varvec{t}}}+{{\varvec{\beta}}}_{10}{\left({\varvec{I}}{\varvec{F}}{\varvec{F}}\right)}_{{\varvec{i}}{\varvec{t}}}+{{\varvec{\beta}}}_{11}{\left({\varvec{S}}{\varvec{A}}{\varvec{B}}\right)}_{{\varvec{i}}{\varvec{t}}}+{{\varvec{\beta}}}_{12}{\left({\varvec{D}}{\varvec{H}}{\varvec{G}}\right)}_{{\varvec{i}}{\varvec{t}}}+{{\varvec{\beta}}}_{13}{\left({\varvec{D}}{\varvec{L}}{\varvec{L}}{\varvec{P}}\right)}_{{\varvec{i}}{\varvec{t}}}+{{\varvec{\beta}}}_{14}{\left({\varvec{C}}{\varvec{A}}{\varvec{L}}{\varvec{D}}\right)}_{{\varvec{i}}{\varvec{t}}}$$

Where $i=20$ and $t=[2004;2021]$ and DCP, RIU and CW10 are the instrumented variables, while MCEC, DRMH, IB, ERSUD, EUSDA, PMA, IFF, SAB, DHG, DLLP and CALD are the instrumental variables (Table 13).

**Table 13:** Instrumental Variable Estimation

	Variables	Acronym	Coefficient	Standard Error	P-value
	Const		17.87	1.56	***
X	Duration of civil proceedings	DCP	0.01	0	***
	Regular internet users	RIU	-0.27	0.04	***
	Companies with at least 10 employees with web sales to end customers	CW10	0.27	0.15	*
Z	Municipalities' current expenditure on culture	MCEC	Statistics		
	Density and relevance of museum heritage	DRMH	Mean Dependent Variable		10.13
	Illegal building	IB	Sum Squared Residual		13728.71
	Erosion of rural space from urban dispersion	ERSUD	R-Squared		0.09
	Erosion of rural space due to abandonment	EUSDA	F(3,351)		20.23
	Pressure from mining activities	PMA	S.D. Dependent Variable		6.31
	Impact of forest fires	IFF	S.E. of Regression		6.25
	Spread of agritourism businesses	SAB	Adjusted R-Squared		0.08
	Density of historic greenery	DHG	P-Value (F)		4.08e-12
	Dissatisfaction with the landscape of the living place	DLLP			
	Concern about landscape deterioration	CALD			

Therefore, the data shows that hospital emigration tends to grow with the growth of civil proceedings, which is obviously a negative fact from a governance point of view, with the reduction of internet users and the growth of companies with at least 10 employees selling online. The data therefore demonstrate that the worsening of some elements of governance can lead to a growth in the level of hospital emigration within the Italian regions in the period considered.

### Aggregate effect of ESG variables on HEAR among Italian regions

In summary we can note that the analysis shows that hospital emigration tends to be inversely associated with the E, S and G components of the ESG model. In fact, as demonstrated by the econometric models even after having controlled for endogeneity through the use of the instrumental variables model, we can see that hospital migration grows if the environment, the social dimension and governance deteriorate. It follows that the Italian regions that have worse scores in terms of ESG are more exposed to the risk of hospital emigration. Our findings indicate that Italian regions with lower ESG scores are more vulnerable to hospital emigration. This trend underscores the importance of improving environmental conditions, social services, and governance to retain residents and reduce the strain on healthcare systems in regions with better ESG scores. Policymakers should focus on enhancing these components to mitigate the risk of hospital emigration. Addressing environmental issues through sustainable practices, improving social infrastructure by investing in healthcare and education, and strengthening governance can help regions retain their populations. Such improvements not only enhance the quality of life for residents but also reduce the economic and social costs associated with hospital emigration.

## Clusterization with k-Means algorithm optimized with the Silhouette coefficient

Below we present a clustering with k-Means algorithm optimized with the Silhouette coefficient. Since the k-Means algorithm is an unsupervised algorithm, it is necessary to identify a criterion that can be used to choose the optimal number of k. In the case presented below, the Silhouette Coefficient was chosen. The Silhouette Coefficient varies from -1 to 1 and assigns a value to each k. The k with a higher Silhoeutte Coefficient value is chosen as the optimal value. In the analyzed case, two clusters composed as indicated below are identified:

- Cluster 1: Sicily, Friuli Venezia Giulia, Tuscany, Veneto, Emilia Romagna, Piedmont, Puglia, Lazio, Sardinia, Campania, Lombardy, Trentino Alto Adige, Umbria, Marche, Liguria;
- Cluster 2: Basilicata, Molise, Calabria, Valle d'Aosta, Abruzzo.

**Fig. 2:**
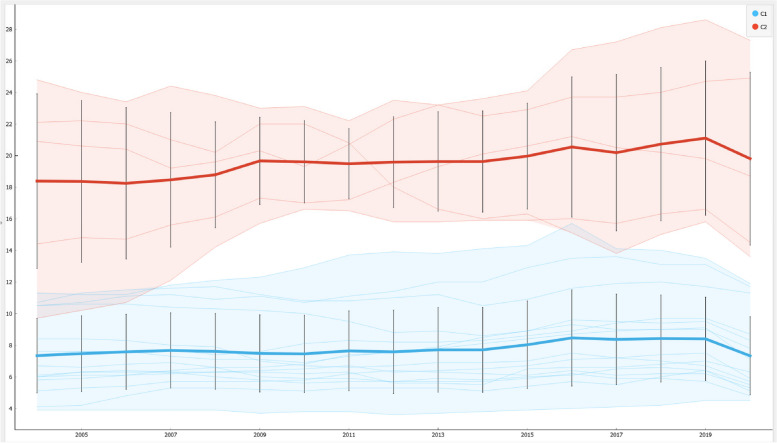
By optimizing the k-Means algorithm with the Silhouette coefficient it is possible to verify that the optimal k value is k=2. By calculating the average of the individual clusters it is possible to verify which of the clusters has a dominant value of the observed variable. In the case presented, the average of Cluster 2 is higher than the average of Cluster 1. The following ordering of the Clusters therefore derives: Cluster 2>Clusters 1

The clusters can be evaluated based on the average value. That is, clusters that have a higher mean value are dominant compared to other clusters. In the case analyzed the average value of cluster 2 tends to be high compared to the value of Cluster 1. It follows that the citizens of Cluster 2 have much higher levels of hospital emigration than the citizens of Cluster 1. It is possible to note that the Cluster 2 is made up of 4 southern regions and the Aosta Valley. The regions that make up Cluster 1 are regions of Central-Northern Italy with the sole exception of Puglia, Sardinia and Campania. However, although clustering with k=2 is to be preferred on the basis of the Silhouette coefficient, it also presents significant limitations from a metric point of view. In fact, 15 Italian regions out of 20, i.e. 75% of the observed sample, are allocated within the same cluster, i.e. Cluster 1. This is a condition that manifests a certain inefficiency as among the 15 regions that are part of the In Cluster 1 there are some that present significant differences from a socio-economic and demographic point of view (Fig. 2).

Therefore, to obtain a result that presents a greater level of redistribution of the regions within the variables it is possible to increase the value of k=3. With k=3 the value of the Silhouette Coefficient is still positive and appears to be the best result after the optimal result of k=2. In fact, while the value of the Silhouette Coefficient with k=2 is equal to 0.640, the value of the Silhouette Coefficient with k=3 is equal to 0.563. Therefore, by setting k=3 it is possible to obtain the following three-cluster structure, namely:

- *Cluster 1*: Tuscany, Friuli Venezia Giulia, Veneto, Emilia Romagna, Sicily, Sardinia, Piedmont, Lombardy, Lazio, Puglia, Campania;
- *Cluster 2*: Basilicata, Molise, Valle d'Aosta, Calabria;
- *Cluster 3*: Liguria, Marche, Umbria, Abruzzo, Trentino Alto Adige.

**Fig. 3:**
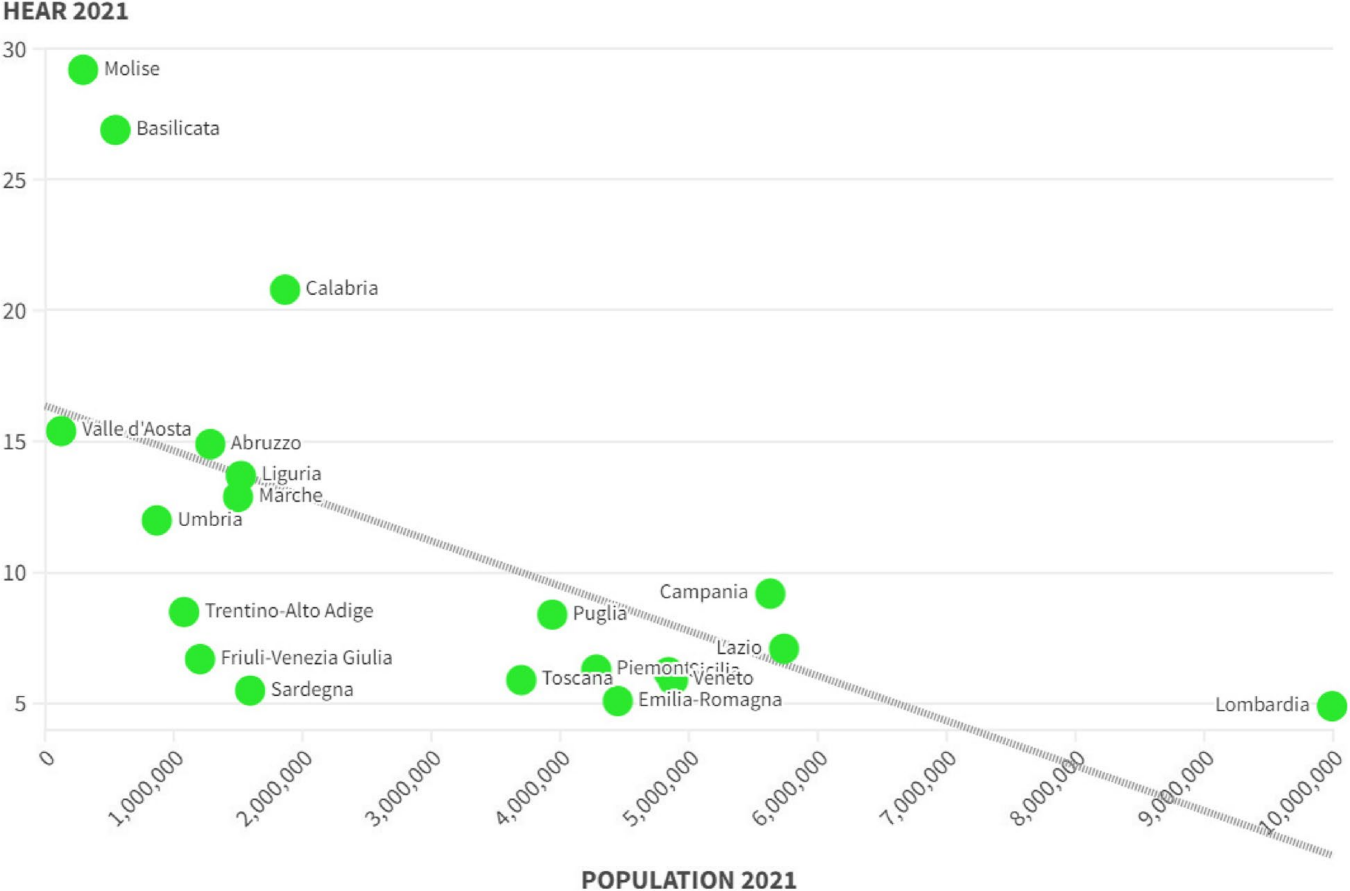
The negative relationship between the value of hospital emigration and the value of the resident population in 2021. Regions that have a smaller population tend to be characterized by a growth in hospital migration

Through the analysis of the average of the clusters it is possible to identify an ordering of the clusters. In the case of k=3 it turns out that Cluster 2>Cluster 3>Cluster 1. The dominant Cluster is Cluster 2 made up of 3 southern regions and the Aosta Valley. Followed by the Cluster, which is very heterogeneous from a geographical point of view, and is made up of both southern regions, such as Abruzzo, and central Italy, such as Marche and Umbria and also northern regions such as Liguria and Trentino Alto Adige. However, we can see that there is a negative relationship between the average population of the clusters and the HEAR value at the cluster level. In fact, by calculating the average of the population of the cluster regions in 2023 it is possible to note that: the average of the Cluster 2 regions is equal to 697,689, the average of the Cluster 3 regions is equal to 1,236,555, and the average of Cluster 1 is equal to 4,534,290. That is, hospital emigration tends to grow with the reduction of the population. Particularly in regions with low populations such as the Cluster 2 regions, the value of hospital emigration tends to be maximum. On the contrary, in regions with a high population, as in the case of Cluster 1 regions, the value of hospital emigration tends to decrease. To verify this intuition it is possible to plot the value of HEAR 2021 against the value of the population of the Italian regions in 2021. The data suggests the presence of a negative relationship between the value of hospital emigration and the population resident in the Italian regions (Fig. 3).

From the analysis of the clustering with the k-Means algorithm, it is therefore clear that there is a condition in the Italian regions based on a significant inequality induced by the differentials of the resident population. That is, regions that have a larger resident population also have greater opportunities and resources to make their healthcare system more efficient with respect to demographic needs. On the contrary, regions that have a smaller population also have fewer possibilities and resources to invest in the regional healthcare system and offer adequate services to residents. It therefore follows that regions that have a population of less than 1.5 million inhabitants tend to be subjected to significant hospital emigration. The only two regions with a population close to 1.5 million inhabitants and which remain within Cluster 1, i.e. the Cluster with the lowest hospital emigration, both in the case of k=2 and in the case of k=3, are Sardinia and Friuli Venezia Giulia. However, it is possible to note that both Sardinia and Friuli Venezia Giulia are two regions with special administrative autonomy. The presence of Sardinia and Friuli Venezia Giulia in the cluster with low hospital emigration, i.e. in Cluster 1, could be due on the one hand to the managerial capabilities of the regional ruling class and on the other hand to the ability to use the opportunities offered by special administrative autonomy.

### Ranking of the regions by value of healthcare emigration in 2021

**Fig. 4:**
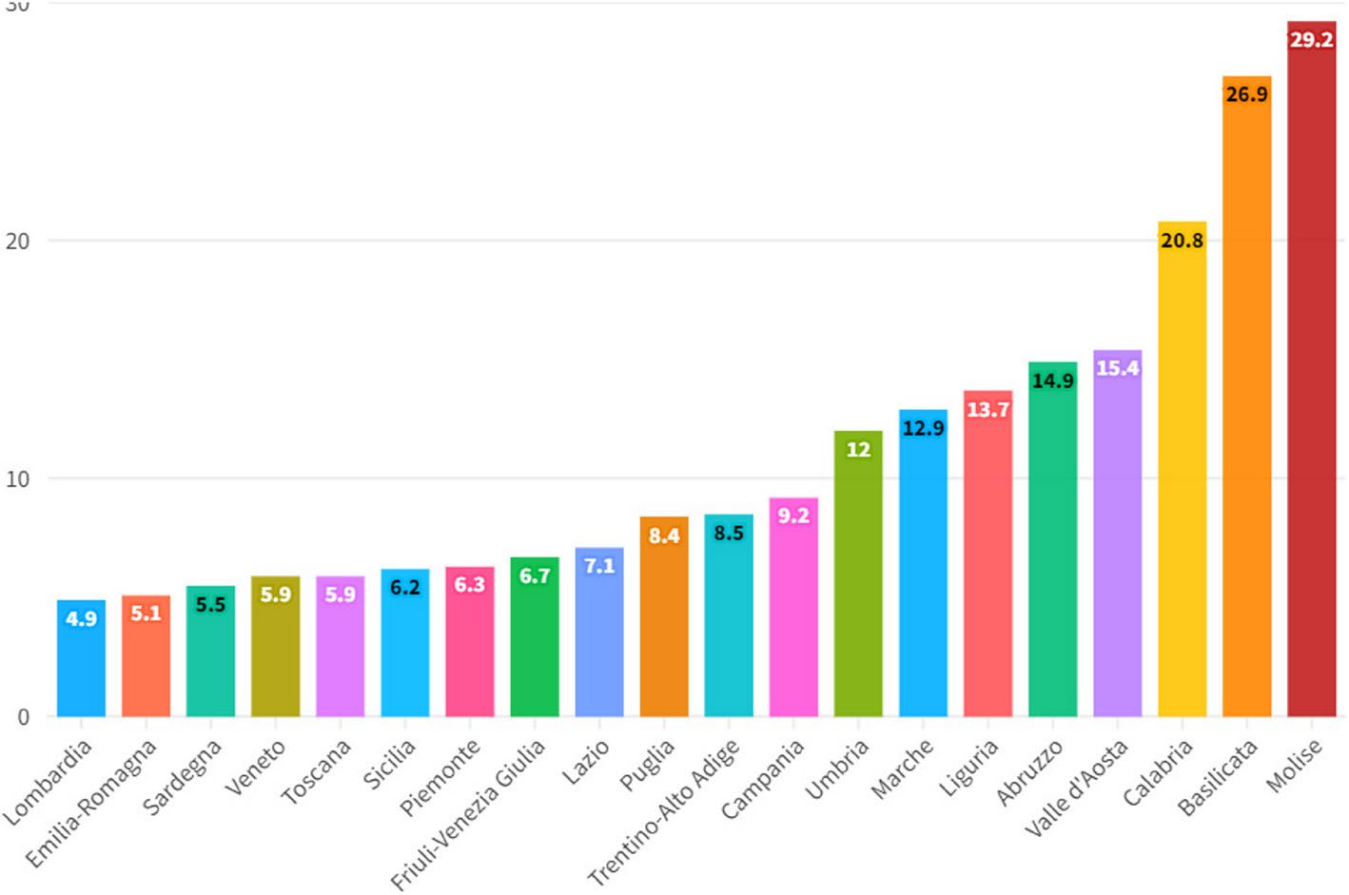
Value of Hospital Emigration in 2021 in the Italian Regions. Source: ISTAT-BES

Molise is in first place by value of healthcare emigration in 2021 with an amount of 29.2 units, followed by Basilicata with 26.9 units and Calabria with 20.8 units. In the middle of the table are Campania with a hospital emigration value of 9.2 units, followed by Trentino Alto Adige with a value of 8.5 and Puglia with an amount of 8.4 units. Sardinia closes the ranking with a value of 5.5 units, followed by Emilia Romagna with an amount of 5.1 units and Lombardy with a value of 4.9 units (Fig. 4).

### Ranking of the Italian regions by percentage change in the value of hospital emigration between 2004 and 2021

**Fig. 5:**
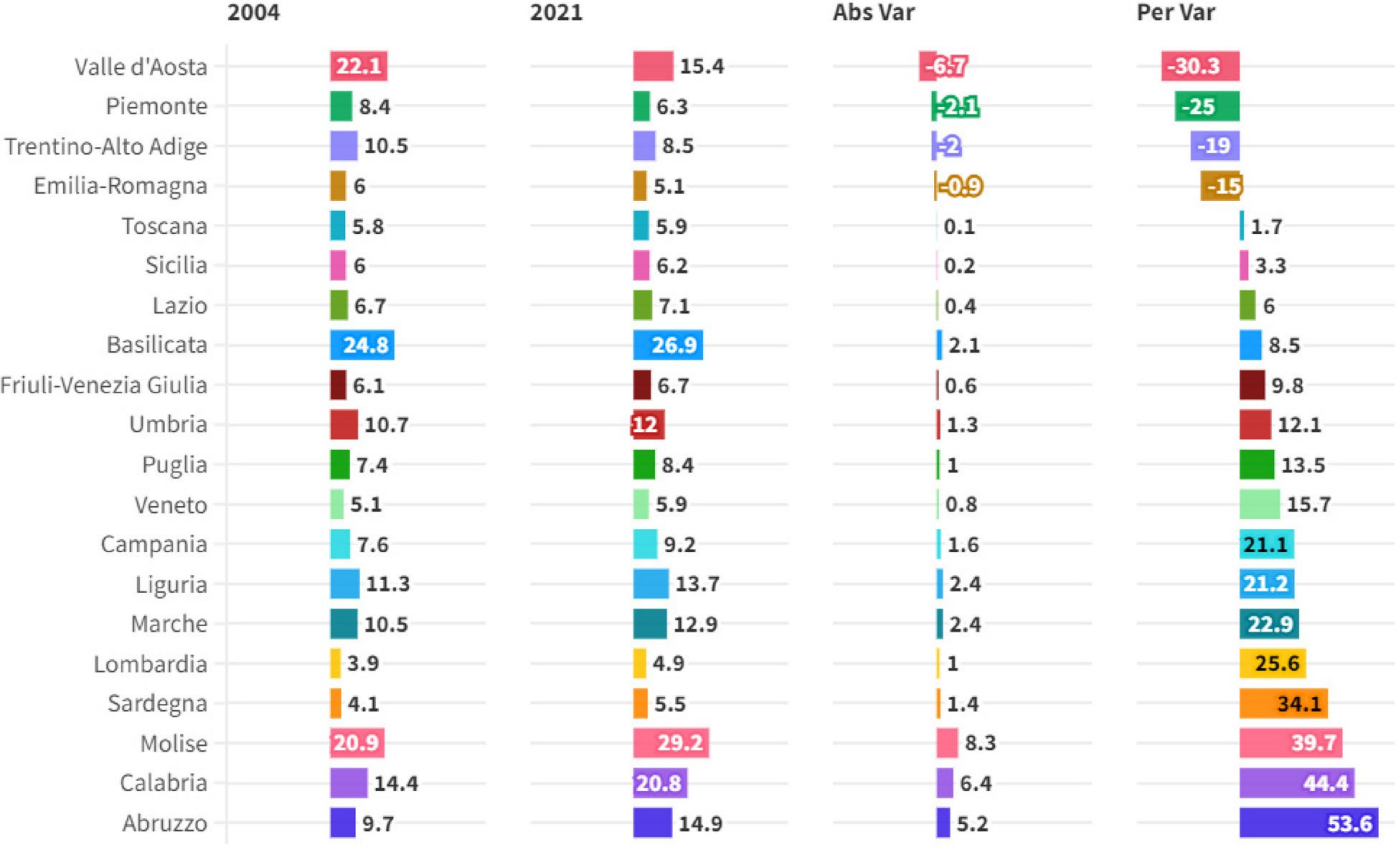
Percentage and absolute change in the value of hospital migration between 2004 and 2021 in the Italian regions. Source: ISTAT-BES

Abruzzo is in first place for the value of the percentage change in hospital emigration with a value equal to +53.60%, going from an amount from 9.70 units in 2004 up to 14.90 units in 2021 or equal to an amount of 5.20 units. Calabria follows with a variation equal to +44.40% corresponding to a variation from an amount of 14.40 units up to 20.80 units or equal to 6.40 units. In third place is Molise with a variation equal to +39.70% corresponding to a variation from an amount of 20.90 units in 2004 up to a value of 29.20 units in 2021 corresponding to an amount of 8.30 unit. In the middle of the table are Veneto with a value of +15.70% corresponding to a variation from an amount of 5.10 units in 2004 up to a value of 5.90 units in 2021 or equal to an amount of 0.80 unit. Puglia follows with an amount equal to +13.50% corresponding to a variation from an amount of 7.40 units in 2004 up to a value of 8.40 units in 2021 or equal to +1.00 units. Umbria is in eleventh place with a variation equal to 12.10% corresponding to a variation from an amount of 10.70 units in 2004 up to a value of 12.00 units in 2021 (Fig. 5).

Trentino Alto Adige closes the ranking with -19.00% corresponding to a variation from 10.50 units in 2004 up to a value of 8.50 units in 2021 or equal to -2.00 units. Piedmont follows with a value of -25.00% corresponding to a variation from an amount of 8.40 units up to a value of 6.30 units. Valle d'Aosta closes the ranking with a variation equal to -30.30% corresponding to a variation from 22.10 units up to 15.40 units or equal to -6.70 units. On average, the value of hospital migration decreased from an amount of 10.10 units to a value of 11.28 units or equal to a value of 1.18 units equal to 11.63%.

## Prediction with machine learning algorithms for the estimation of the future value of hospital migration

**Fig. 6:**
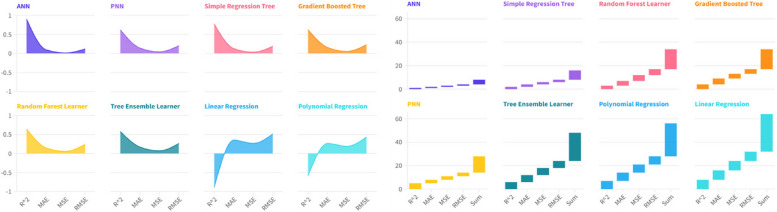
Statistical Measures for the evaluation of the best predictive machine learning algorithm

Below we present an analysis through the application of machine learning algorithms for predicting the future value of hospital migration. The algorithms were trained using 70% of the data while the remaining 30% was used for the actual prediction. The algorithms are analyzed through performance analysis from a statistical point of view, i.e. maximization of the R^2 and minimization of Mean Absolute Error-MAE, Mean Squared Error-MSE, and Root Mean Squared Error-RMSE. The statistical indicators were calculated as follows:

- R Squared $$={R}^{2}=1-\frac{SumSquaredRegression}{TotalSumOfSquares}=1-\frac{\sum {\left({y}_{i}-{\widehat{y}}_{i}\right)}^{2}}{\sum {\left({y}_{i}-{\overline{y} }_{i}\right)}^{2}}$$
- Mean Average Error $=MAE=\frac{\sum_{i=1}^{n}|{y}_{i}-{\widehat{y}}_{i}|}{n}$
- Mean Squared Error=$MSE$=$\frac{1}{n}\sum_{i=1}^{n}{\left({y}_{i}-{\widehat{y}}_{i}\right)}^{2}$
- Root Mean Squared Error=$RMSE$=$\sqrt{\frac{1}{n}\sum_{i=1}^{n}({y}_{i}-\widehat{{y}_{i}})^2}$

where ${{\varvec{y}}}_{{\varvec{i}}}$ is the true value, $\widehat{{\varvec{y}}}=$ predicted value, and $\overline{{\varvec{y}} }=\frac{\sum {\varvec{y}}}{{\varvec{n}}}$, **n**=sample size (Fig. 6).

Each algorithm is given a vote within the ranking of the statistical indicators analysed. The individual placements within the individual rankings are added. The algorithm that shows lower levels in terms of overall payoff also turns out to be the most efficient algorithm from a predictive point of view. The following ordering of the clusters is then identified:

- ANN-Artificial Neural Network with a payoff value of 4;
- Simple Regression Tree with a payoff value of 8;
- PNN-Probabilistic Neural Network with a payoff value of 14;
- Random Forest and Gradient Boosted Tree with a payoff value of 17;
- Tree Ensemble with a payoff value of 24;
- Polynomial Regression with a payoff value of 28;
- Linear Regression with a payoff value of 32.

A synthesis of the main results is indicated in the Table 14.

**Table 14:** Ranking of Algorithm based on R-Squared and Statistical Errors

Rank	Algorithm	R^2	Rank	Algorithm	MAE
1	ANN	0.898	1	ANN	0.095
2	Simple Regression Tree	0.774	2	Simple Regression Tree	0.127
3	Random Forest	0.649	3	PNN	0.141
4	Gradient Boosted Tree	0.623	4	Random Forest	0.147
5	PNN	0.616	5	Gradient Boosted Tree	0.157
6	Tree Ensemble	0.576	6	Tree Ensemble	0.171
7	Polynomial Regression	-0.583	7	Polynomial Regression	0.265
8	Linear Regression	-0.886	8	Linear Regression	0.352
Rank	Algorithm	MSE	Rank	Algorithm	RMSE
1	ANN	0.013	1	ANN	0.116
2	Simple Regression Tree	0.034	2	Simple Regression Tree	0.186
3	PNN	0.042	3	PNN	0.205
4	Gradient Boosted Tree	0.053	4	Gradient Boosted Tree	0.230
5	Random Forest	0.056	5	Random Forest	0.238
6	Tree Ensemble	0.071	6	Tree Ensemble	0.266
7	Polynomial Regression	0.190	7	Polynomial Regression	0.436
8	Linear Regression	0.264	8	Linear Regression	0.514

**Fig. 7:**
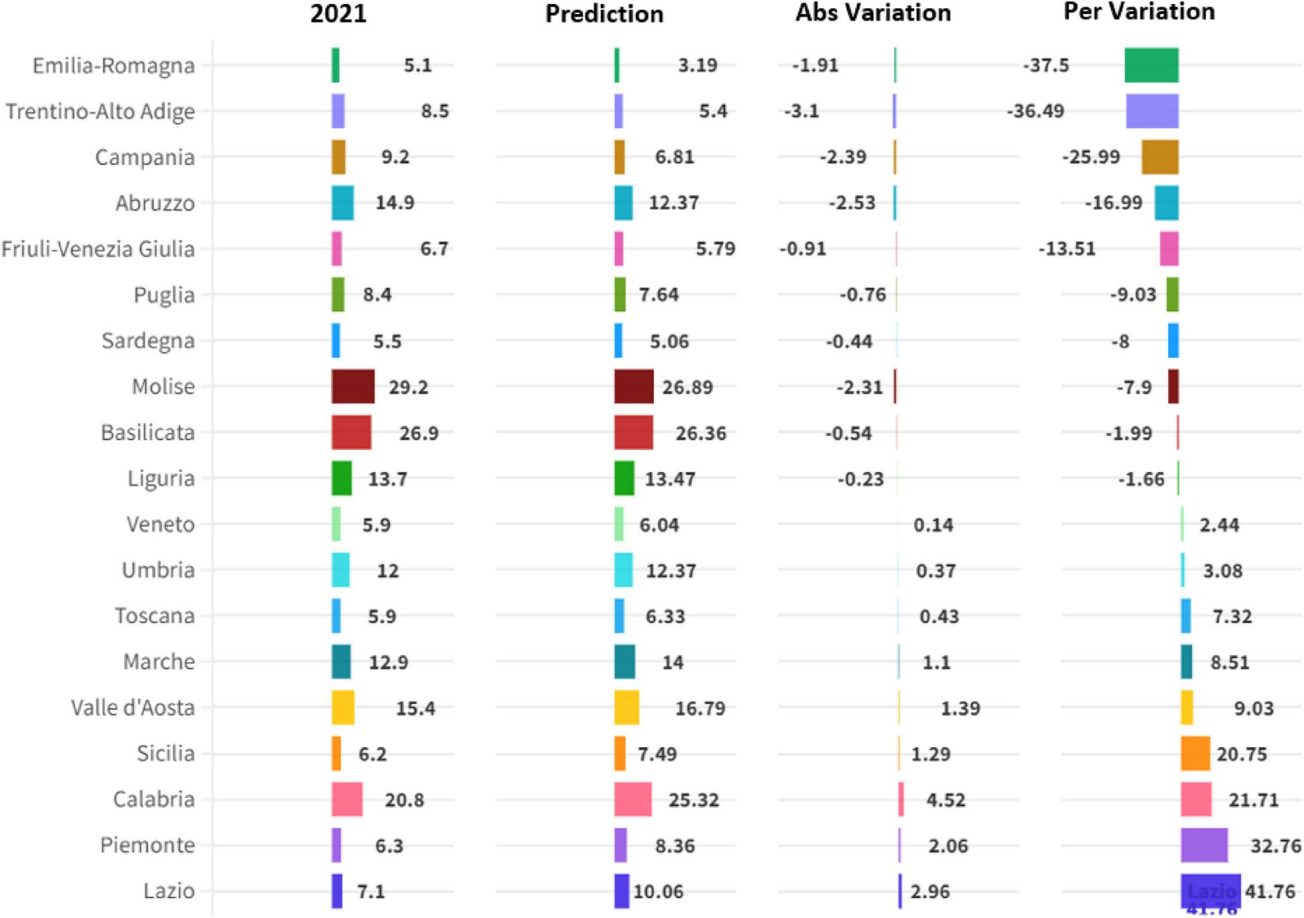
Summary of the productions and of the absolute and percentage variations of the predicted values with the ANN-Artificial Neural Network algorithm

Therefore, by applying the ANN-Artificial Neural Network algorithm it is possible to predict the future trend of the variable analysed or relating to hospital emigration. From the point of view of predictions we can note that there are regions for which a growth in the value of hospital emigration is expected and regions for which a reduction in hospital emigration is instead expected. Hospital emigration is predicted to grow in Lazio with a value equal to 41.76% corresponding to a variation from an amount of 7.1 units up to a value of 10.06 units or equal to a variation of 2.96 units. Followed by Piedmont with +32.76%, Calabria with +21.71%, Sicily with +20.75%, Valle d'Aosta with a value of 9.03%, Marche with 8.51%, Tuscany with 7, 32%, Umbria 3.08%, Veneto 2.44%. The regions in which a reduction in the value of hospital migration is predicted are: Liguria with -1.66%, Basilicata with -1.99%, Molise with -7.9%, Sardinia with -8.00%, Puglia with - 9.03%, Friuli Venezia Giulia with -13.51%, Abruzzo with -16.99%, Campania with -25.99%, Trentino Aldo Adige with -36.49%, Emilia Romagna with -37.5% (Fig. 7).

### Critical analysis of predictions

Analysing the predictions produced by the algorithm it is necessary to ask ourselves whether they actually make sense when compared to the real conditions of the Italian regions in terms of hospital healthcare provision. For example, it is very unlikely that the value of hospital emigration will decrease in Molise. In fact, Molise is one of the small regions that have very high levels of hospital emigration and this value will probably continue to be high in the future. Another predicted value that appears difficult to justify is Calabria. In fact, for Calabria the ANN algorithm predicts growth from an amount of 20.8 units up to a value of 25.32 units or a growth of 21.71%. Calabria is one of the Italian regions with the highest level of hospital emigration and although this value will certainly remain high in the future, it is very unlikely that it will grow by 21.71% as predicted by the ANN algorithm. A further case to be critically analyzed is Campania: the value of hospital emigration is predicted to decrease by 25.99%, going from 9.2 to 6.81. It is very unlikely that the reduction in hospital emigration will decrease by 25.99%, especially due to economic policies that tend to reduce the amount of healthcare spending as a percentage of GDP. For similar reasons, it is very unlikely that there will be a reduction in the value of hospital emigration in Trentino Alto Adige and Emilia Romagna. For these regions the algorithm predicts a reduction of 36.49% for Trentino Alto Adige and 37.50% for Emilia Romagna respectively. Finally, the average value of hospital emigration among the Italian regions is predicted to decrease even if at a marginal level or equal to -0.39%. However, it is very likely that there will be a growth in the average value of hospital emigration in the Italian regions due to the reduction in healthcare spending which could generate a further growth in the gap between Northern and Southern Italy in terms of distribution of public resources and access to the system healthcare. Therefore, it is necessary to underline that the ANN algorithm is the best predictor based on the metric indicators presented. However, the results obtained must be subjected to further qualitative analyses and must also be interpreted in light of health economic policies which are increasingly oriented towards reducing public spending as a percentage of GDP. In fact, considering the trend of Italian public spending, it is very probable that the value of hospital emigration in the Italian regions will grow significantly in the future.

## The international relevance of the analysis of hospital emigration across Italian regions

The analysis conducted on hospital emigration in Italy is certainly useful for analyzing the condition of the Italian regions, but it can also be relevant from an international point of view. In fact, Italy is a country characterized by a significant heterogeneity of the regions. The regions of Central-Northern Italy are much richer and more advanced from an economic and technical-scientific point of view than the southern regions. This disparity, often referred to as the North-South divide, profoundly affects various aspects of life in Italy, including healthcare. The Northern regions tend to offer much better health services than the regions of Southern Italy. This dynamic is crucial to understand, especially in light of the proposed reforms by the conservative and right wing government led by the post-fascist Giorgia Meloni, which could potentially exacerbate these disparities. The Northern regions of Italy, with their advanced infrastructure, better-funded hospitals, and access to cutting-edge medical technologies, attract patients from the South seeking superior medical care. The economic and technical-scientific advancements in these regions translate into higher quality healthcare services. Northern hospitals often feature state-of-the-art facilities, highly specialized medical professionals, and shorter waiting times for treatments. Conversely, the Southern regions struggle with underfunded healthcare systems, outdated facilities, and a shortage of medical professionals. This issue is not unique to Italy. Hospital emigration can be generalized internationally as many countries exhibit significant regional disparities. These disparities often correlate with variations in healthcare quality and accessibility, prompting patients to travel from less affluent regions to more developed areas within the same country. In fact, Italy is not the only country characterized by the presence of significant regional gaps. In addition, other countries with a medium-high per capita income and with a high per capita income have very similar problems compared to the Italian ones. For example, the United Kingdom is characterized by a divide between Scotland and England. The inhabitants of Scotland have lower per capita incomes than those of England and this condition could also generate effects in terms of hospital migration. In the UK, healthcare is provided by the National Health Service (NHS), which, despite being a unified system, experiences regional variations in quality and access. England, particularly London and the South East, benefits from better-funded hospitals and more comprehensive healthcare services compared to Scotland and other regions. This discrepancy can lead to hospital emigration, where patients from Scotland might seek treatment in English hospitals. Similarly, a significant regional divide exists in the United States. There is a considerable gap between US states in terms of per capita income. For example, Mississippi, West Virginia, and Arkansas have per capita incomes that are equal to about half or 40% of the wealthiest US states such as New York, Massachusetts, and Washington. This economic divide extends to healthcare, where wealthier states have better hospitals, more medical professionals, and advanced medical technologies. As a result, residents of poorer states may travel to wealthier states to receive better medical care, creating a pattern of hospital emigration. The healthcare system in the USA, being largely privatized, amplifies these disparities as states with higher incomes can invest more in healthcare infrastructure and services. Germany also presents a similar scenario. Despite being one of the wealthiest countries in Europe, it has significant regional disparities. Regions such as Mecklenburg-Vorpommern, Thuringia, and Saxony-Anhalt have incomes equal to approximately half of the richest per capita income regions such as Bavaria, Bremen, and Hamburg. These economic differences are reflected in healthcare services, where wealthier regions offer superior medical facilities and treatments. Consequently, residents from less affluent regions may seek healthcare in more developed areas, contributing to hospital emigration within Germany. It therefore follows that hospital emigration within the regions of a state is a widespread phenomenon that can also be detected for states that have high per capita incomes if it is possible to detect the presence of significant regional gaps in terms of per capita income. The healthcare system is inherently expensive in terms of spending. Therefore, regions with higher per capita incomes have a greater ability to invest in new technologies and scientific research in the medical and healthcare sector. This investment translates into better healthcare services, attracting patients from less affluent regions. Moreover, the phenomenon of hospital emigration is not only a matter of seeking better medical treatment but also involves other factors such as shorter waiting times and access to specialized care. Patients from less developed regions often face long waiting periods for certain treatments, prompting them to seek quicker alternatives in more developed areas. Additionally, specialized medical treatments and procedures, which are often available only in advanced healthcare facilities, attract patients from regions where such services are lacking. The results of our study on hospital emigration in Italy can be extrapolated to other countries with similar regional disparities. Countries with medium-high per capita income, such as the UK, USA, and Germany, exhibit significant regional economic gaps that influence healthcare quality and access. These gaps drive hospital emigration, as patients from less affluent regions travel to wealthier areas for better medical care. In conclusion, the analysis of hospital emigration in Italy not only sheds light on the regional disparities within the country but also provides a framework for understanding similar phenomena in other countries. Regional economic disparities significantly affect healthcare quality and access, leading to hospital emigration. This trend is observed not only in Italy but also in countries like the UK, USA, and Germany. Addressing these disparities requires targeted policies to improve healthcare infrastructure and services in less affluent regions, thereby reducing the need for patients to seek medical care elsewhere. The findings from Italy can inform international efforts to tackle regional healthcare inequalities, ensuring more equitable access to quality medical services for all citizens.

## Conclusions

This article addressed the issue of hospital migration in Italian regions in the context of the ESG model inspired by SDGs policies. A complex econometric analysis was presented aimed at first verifying the impact of the individual components E, S and G in determining the level of patient migration. Subsequently, the aggregate impact of ESG in determining HEAR was also calculated. The results show that hospital migration tends to be inversely related to ESG factors. Therefore, regions that have high ESG scores also tend to have a low level of patient migration. Conversely, regions that have low levels of ESG tend to have higher patient migration. Subsequently, clustering was carried out with the k-Means algorithm to capture the elements of the regional distribution of patient migration. The results show a significant inequality between the southern regions and the northern regions. That is, the southern regions tend to have higher levels of patient migration than the northern regions. However, some role must also be recognized to demographic aspects: that is, sparsely populated regions tend to have increasing levels of patient migration. Finally, a prediction is presented by comparing eight different machine learning algorithms evaluated based on their ability to maximize the R-squared and minimize the value of statistical errors. The analysis shows that the most efficient algorithm is the ANN. The ANN predicts a reduction in patient migration. However, in the discussion of the results this prediction is contested as unlikely to be probable, not for metric-statistical reasons, but rather for political-institutional motivations. In fact, the political economic conditions and institutional reforms that could lead to fiscal independence of the North-East regions compared to the rest of Italy could lead to a significant growth in hospital migration from South to North. It is therefore necessary for the policy maker to take considering the possibility of reorganizing healthcare in order to guarantee access to healthcare while reducing regional inequalities.

## Data Availability

The datasets generated and/or analysed during the current study are available in the ISTAT-BES repository, link: https://www.istat.it/statistiche-per-temi/focus/benessere-e-sostenibilita/la-misurazione-del-benessere-bes/gli-indicatori-del-bes/.
